# Integrative Structural Characterization of *Candida glabrata* Phosphoglycerate Kinase by Small-Angle X‑ray Scattering and AlphaFold: Implications for Therapeutic Targeting in Candidiasis

**DOI:** 10.1021/acsomega.5c11751

**Published:** 2026-01-21

**Authors:** Mayra Cuéllar-Cruz, Edson E. Maqueda Cabrera, Dritan Siliqi, Abel Moreno

**Affiliations:** † Departamento de Biología, División de Ciencias Naturales y Exactas, Campus Guanajuato, 14654 Universidad de Guanajuato, Noria Alta S/N, Col. Noria Alta, C.P., 36050 Guanajuato, Guanajuato, Mexico; ‡ Istituto di Cristallografia, 296400 Consiglio Nazionale delle Ricerche, Via G. Amendola 122/O, 70126 Bari, Italy; § Instituto de Química, 7180 Universidad Nacional Autónoma de México, Av. Universidad 3000, Ciudad Universitaria, Ciudad de México 04510, Mexico

## Abstract

*Candida glabrata* is the second leading cause of mortality in immunocompromised patients hospitalized for invasive candidiasis (IC). Several drugs have been available to treat this disease for decades, such as polyenes, azoles, echinocandins, flucytosine, and, in critical cases, amphotericin B. However, these antifungals’ constant and routine use have led to the development of resistance mechanisms, making the design and development of new drugs indispensable. The first step for the design and subsequent synthesis of a new chemical molecule as a potential antifungal is the identification of new therapeutic targets. In that pathway, our working group has identified moonlight-like cell wall proteins (CWPs) in different *Candida* species that can act as potential antifungal targets. One of these moonlight-like CWPs is phosphoglycerate kinase (Pgk) from *C. glabrata*. Once Pgk was identified as a potential therapeutic target in different human pathogens, the first step to perform drug design against this moonlight-like CWP was the elucidation of the three-dimensional (3D) structure since the 3D structure is key to understanding the interactions between a drug candidate and its target at the molecular level. In the present work, we aimed to elucidate the 3D structure of *C. glabrata* Pgk. To elucidate the 3D structure of this protein, the recombinant protein was expressed, purified, and structurally resolved by means of a structural analysis by small-angle X-ray scattering (SAXS). Additionally, in order to evaluate its potential as a therapeutic target, we have performed molecular docking studies and enzymatic activity assays with pure Pgk using known antifungals amphotericin B, nystatin, and fluconazole and with the new plausible drugs, such as nilotinib and netupitant. Our results showed some similarities and differences with orthologous Pgk proteins from other organisms, which was expected since Pgk has been observed to have evolved in the kingdoms of life. Molecular docking studies showed that Pgk interacts with all of the compounds tested. In enzyme activity assays, a change in the kinetic parameter Km on the enzyme Pgk was observed in response to its interaction with nilotinib, netupitant, and amphotericin B. Thus, our results allow us to propose Pgk from *C. glabrata* as a possible therapeutic target against candidiasis. We consider it essential to design and develop new molecules specifically targeting this enzyme, which will contribute to a decrease in mortality associated with IC and improve the patient’s quality of life.

## Graphical abstract

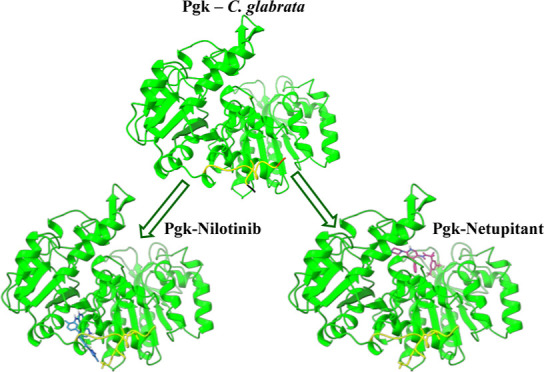

## 1 Introduction

Invasive fungal infections associated with the pathogenic species of the genus *Candida* are the leading cause of annual deaths of patients hospitalized in intensive care units (ICUs).^1,2^ The pathogenic species identified in patient samples are *Candida albicans*, followed by *Candida glabrata*, *Candida krusei*, *Candida parapsilosis*, *Candida Tropicalis,* and recently *Candida auris*. Invasive candidiasis (IC) affects the bloodstream (candidemia) and various organs, such as liver, kidney, brain, heart, eyes, spleen, lungs, digestive system, skin, and bones.^3,4^ In the last three decades, there has been an increase in the incidence of IC due to treatments related to ICU hospitalization, patient health condition, breakdown of skin, or gastrointestinal barriers due to inflammation, prolonged, or repeated administration of broad-spectrum drugs, as well as the biofilm-forming capacity of *Candida* species. Together, these factors have favored resistance to antifungals.^5–7^ Thus, as IC is a multifactorial disease, treatment is not always effective for all patients. Even though different groups of drugs are currently available for the treatment of IC, early diagnosis of the disease, clinical evaluation of the patient should be considered,^4,8^ as well as the determination of the *Candida* species causing the condition, since there are species that are innately resistant to azoles, so the correct identification of the *Candida* species, in conjunction with the other factors allows administering the most effective systemic antifungal therapy possible.^9,10^ Among the antifungals routinely administered against IC are polyenes, azoles, echinocandins, 5-fluorocytosine (flucytosine), and in critical cases, amphotericin B. However, even though these drugs are currently routinely used, they have been used to treat IC for six decades.^11–13^ This has led to the development of resistance mechanisms by different *Candida* species and/or strains,^14–17^ as well as nephrotoxicity and patient intolerance.^18,19^ Thus, most therapies against this disease have become increasingly ineffective due to the rise of drug-resistant strains. The development of new antifungals is a challenge, since they must comply with the principle of selective toxicity proposed by Paul Ehrlich, which is that they must effectively eliminate the microorganisms without harming the host cells.^20^ *Candida* species are eukaryotic cells like humans, but with the great difference that these pathogens have a cell wall (CW), while human cells do not. This is a great advantage in designing potential antifungals against molecules present in the CW. In our work group, we have a special interest in identifying potential therapeutic targets in *Candida* CW, against which we can design new chemical molecules that allow a new alternative treatment against IC. In this pathway, we have identified CW proteins (CWPs) in different virulence and/or pathogenicity factors in *C. albicans*, *C. glabrata*, *C. krusei*, and *C. parapsilosis*.^21–24^ The CWPs we identified correspond to moonlight-like proteins, which, unlike the other CWPs, are not covalently bound to the CW and possess dual function and localization.^25,26^ These characteristics of the moonlight-like CWPs that we identified in response to various virulence factors make them of special relevance because they are immunodominant proteins, which ensures that the treatment developed against them will generate an effective immune response, making them promising candidates in the treatment against IC. Among the 30 moonlight-like CWPs identified in the different conditions and the four *Candida* species, some of them are of special interest, such as phosphoglycerate kinase (Pgk), which was differentially expressed in all virulence factors and all *Candida* species evaluated, making it a potential therapeutic target against CI.^21,23,24^ The canonical function of Pgk is adenosine 5′-triphosphate (ATP) generation in central metabolic pathways.^27,28^ It is a monomeric protein with a molecular weight of approximately 45 kDa.^29^ Among the functions associated with Pgk as moonlight-like CWP are several;^30^ one of these is as an immunomodulator, since in several studies it has been shown to play an important role in disease control, being a possible therapeutic target for drugs to combat various ailments.^31–33^ It has been proposed as a therapeutic target in pathogens of clinical interest, such as *Trypanosoma brucei*, *Trypanosoma cruzi*, and *Leishmania* spp,^30,34–37^ as well as in pathogenic microorganisms such as species of the genus *Streptococcus*.^38^ Once Pgk was identified as a potential therapeutic target in different human pathogens, the first step to perform drug design against this moonlight-like CWP was the elucidation of the three-dimensional (3D) structure, since the 3D structure is key for this purpose, thus also allowing us to understand how the protein performs its function inside the cell and how it interacts with other molecules. In this regard, Buchner was the first to isolate and crystallize a Pgk from yeast extracts.^39,40^ Subsequently, the first low-resolution structures of Pgk from horse muscle^41^ and yeast were achieved.^42^ Thus, Pgk structures in the apo form, such as holo from various organisms, are now available in the NCBI database.^43^ However, although many 3D Pgk structures from various organisms are currently available, the 3D Pgk structure of one of the *Candida* species associated with high morbidity and mortality rates in the IC, *C. glabrata*, has not yet been elucidated. Although this pathogen is the second leading cause of IC worldwide, unlike *C. albicans*, it is innately resistant to azoles, which makes it difficult to treat. In the present work, we aimed to elucidate the 3D structure of Pgk of *C. glabrata*. Having the 3D structure of Pgk of *C. glabrata* will allow the design of chemical molecules against this moonlight-like CWP, which will contribute to a specific treatment of IC, favoring a better quality of life for the patient and a decrease in morbidity and mortality due to this disease. To elucidate the 3D structures of this protein, recombinant protein expression, purification, and structural resolution were carried out through structural analysis by small-angle X-ray scattering (SAXS). Additionally, in order to evaluate its potential as a therapeutic target, we carried out molecular docking and evaluation of the enzymatic activity on the pure Pgk using known antifungals amphotericin B, nystatin, and fluconazole including the new plausible compounds nilotinib and netupitant.

Our results showed some similarities and differences with orthologs Pgk proteins from other organisms, which was expected, as Pgk has been observed to have evolved in the kingdoms of life. Molecular docking studies showed that Pgk interacts with the antifungals tested. In enzyme activity assays, a change in the *V*_max_ and *K*_m_ of Pgk was observed concerning the Pgk-nilotinib and Pgk-netupitant interactions. Nilotinib caused the greatest increase in *K*_m_, followed by netupitant. Thus, our results allow us to propose Pgk from *C. glabrata* as a therapeutic target against candidiasis. We consider it essential to design and develop new drugs against this enzyme, which will contribute to a decrease in mortality associated with IC and improve the patient’s quality of life.

## 2 Experimental Section

### 2.1 Strains and Culture Conditions

The strain used in this study was the so-called pET19b-PGK-Cg (previously reported by Medrano-Díaz^22^), which corresponds to the *Escherichia coli* (*E. coli*) strain BL21 (DE3), transformed with the pET19b vector with the open reading frame of the *PGK* gene from *C. glabrata*, Amp^R^. *E. coli* cells were grown in the Luria–Bertani (LB) medium at pH 7.0, which contains: peptone biotryptase, 10 g/L; NaCl, 5 g/L; yeast extract, 5 g/L. For solid LB, 2% bacteriological agar was added. For selection assays, kanamycin (Kn) 10 μg/mL and/or ampicillin (Amp) 100 μg/mL were added to the medium. The liquid cultures were incubated with orbital shaking at 250 rpm and 37 °C.

### 2.2 Design and Construction of Plasmid for Overexpression of Pgk from *C. glabrata*

Pgk from *C. glabrata* has a size of 417 amino acids. Codon optimization was considered in the design because it is different in *E. coli* concerning *C. glabrata*. The sequence of the optimized PGK gene was sent for synthesis to the GenScript company (Piscataway, New Jersey, USA) according to the original sequence of the protein (reference sequence of *C. glabrata* CBS138 in the *Candida* Genome Database). Restriction sites recognized by the enzymes *XhoI* and *Bam*HI at the 5′ and 3′ ends, respectively, were added to the synthesized sequences in order to subclone the gene sequence into the expression vector pET19b, which adds a 6-histidine tag (His6X) to the recombinant protein. The optimized gene sequences were received inserted into the pET19b expression plasmid.^22^

### 2.3 Obtaining Recombinant Strains

Competent cells were prepared according to the protocol previously described by Medrano-Díaz.^22^ Briefly, a test tube was inoculated with 2 mL of LB + *Cm* medium with *E. coli* BL21 cells and incubated overnight at 37 °C with constant shaking. After incubation had elapsed, 25 mL of LB + *Cm* medium was inoculated with 500 μL of preinoculum of each CmR strain. OD_600 nm_ was read in a spectrophotometer (Thermo Scientific Genesys 20) until an OD_600 nm_ of 0.2–0.5 was reached. Subsequently, the culture was cooled on ice for 10 min together with CaCl_2_/TrisHCl solution pH = 8.0 and after preinoculating the falcon tubes on ice, they were centrifuged 5 min at 4 °C (4000 rpm) and the supernatant was discarded. Subsequently, *E. coli* BL21 *Cm* ^R^ cells were resuspended at a ratio of 1/15 of the original volume (∼1.5 mL) in CaCl_2_/Tris–HCl at pH 8.0. Once 200 μL aliquots of each strain were prepared in Eppendorf tubes, 0.5 μL of the plasmid (Pgk) were added, incubated on ice for 30 min, then placed 2 min at 42 °C in water bath, tubes were quickly transferred to an ice bath for 2 min, 1 mL of sterile LBAmp medium was added, to incubate subsequently at 37 °C with constant shaking for 45 min–1.5 h. The bacterial suspension was cultured on LBAmp agar plates and incubated at 37 °C from 18 to 20 h. Then, some transforming colonies grown in the culture medium were randomly selected in order to verify that the transformed clones contained the PGK gene for which plasmid DNA extraction was performed. Plasmid DNA was isolated by using the Quick Plasmid Miniprep kit (Invitrogen). The pure DNA was digested with Xho1 and BamH1 enzymes according to the supplier’s instructions (Bio-Rad). Digestion products were analyzed on a 0.8% agarose gel by processing the samples as follows: 6 μL of loading buffer ∼ 7 μL of the digested samples, adding 1.0 μL molecular weight marker, 12 μL control sample, loaded, and ran the gel at 90 V. The weight marker molecular weight 1 Kb DNA Ladder (Invitrogen) was used as a reference. Gels were analyzed on a UV light transilluminator on a GelDoc XR image analyzer system (Bio-Rad). Finally, each of the strains whose amplified size corresponded to the restriction-checked full gene size was reseeded in the liquid LB medium and subsequently transferred to sterile cryopreservation vials with sterile 15% glycerol; the cryovials of each recombinant strain were stored at −80 °C. They were also reseeded in the solid LB medium with the antibiotic required for each transformant strain, and these were used for induction of protein expression as described in the following section.

### 2.4 Overexpression of Pgk

Taking as a reference the results obtained by Diaz-Medrano,^22^ for the expression of the recombinant Pgk protein of *C. glabrata*, an isolated colony of the strain was seeded in 5 mL of LB medium supplemented with kanamycin (33 μg/mL), which was incubated at 37 °C with a constant agitation of 250 rpm overnight. Two milliliters of the preinoculum was taken to seed a 50 mL culture of LB medium supplemented with kanamycin (33 μg/mL), incubated at 37 °C with constant 250 rpm agitation until an OD_600 nm_ of 0.5–0.8 was reached. An aliquot was taken corresponding to time zero. The rest of the sample was supplemented with isopropyl-β-D-1-thiogalactopyranoside (IPTG, Promega, Madison, WI) at a concentration of 0.5 mM and incubated at 250 rpm at 37 °C, and aliquots were taken at 6 h of induction.^22^ Cells were collected by centrifugation at 4500 rpm, at 4 °C for 10 min, and washed thrice. The cell pellet was resuspended in lysis buffer (50 mM Tris–HCl, 300 mM NaCl, 10 mM imidazole, 0.03% Tween 20, pH 7.5). Cells were lysed by sonication at 4 °C for 4 min total with intervals of 10 s ON, 40 s OFF, and 6 W power, to 90% amplitude using a Vibra-Cells VCX 130PB sonicator (Sonics, Newtown, CT). The homogenate was centrifuged for 15 min at 12,000 rpm, at 4 °C, and the supernatant (soluble fraction) was separated from the insoluble (pellet). Aliquots of 20 μL of the soluble and insoluble fractions were taken from the lysates of each strain and treated with 5X sample buffer; the molecular weight marker (GoldBio) was used as a reference. All aliquots were subjected to sodium dodecyl sulfate-polyacrylamide gel electrophoresis (SDS-PAGE) on a 12% polyacrylamide gel in a miniProtean II chamber (BioRad) for 1 h at 120 V, to evaluate the induction as well as the purity of the protein obtained.^44^

### 2.5 Purification of Recombinant Pgk Protein

Protein purification was performed according to the protocol previously described by Maqueda-Cabrera,^45^ with modifications. Briefly, a 1 mL HisTrap HP column (GE Healthcare) was used which was equilibrated with equilibration buffer (Na_2_HPO_4·_7H_2_O 50 mM, NaCl 500 mM, imidazole 500 mM), with a total of five volumes at a constant flow rate of 0.5 mL/min with a pressure of 0.5 MPa. Subsequently, 1 mL of the sample was charged into the column with a flow rate of 0.5 mL/min at a pressure of 0.5 MPa. The column was then washed with the binding buffer (Na_2_HPO_4_ 7H_2_O 50 mM, NaCl 500 mM, imidazole 5 mM, PMSF 1 mM, pH 8.0). Subsequently, washing was performed using the same equilibration solution, but 5% glycerol was added (Na_2_HPO_4·_7H_2_O 50 mM, NaCl 500 mM, imidazole 500 mM, glycerol 5%). Elution was then performed with elution buffer containing Na_2_HPO_4._7H_2_O 50 mM, NaCl 500 mM, imidazole 500 mM, PMSF 1 mM, glycerol 5%, pH 8.0, at a flow rate of 0.5 mL/min at a pressure of 0.5 MPa. Protein elution was done with a continuous gradient, and 1 mL fractions (20 drops) were collected. The fractions were collected and preserved for subsequent analysis by SDS-PAGE. The protein was quantified (Bio-Rad protein assay).

### 2.6 Solubility Testing of Pure Recombinant Pgk and Verification by Dynamic Light Scattering Assay

In order to ensure the solubility of Pgk, different tests were carried out with different buffers, resulting in the buffer Tris–HCl 20 mM pH 7.97 and 100 mM NaCl, where the protein showed complete solubility. Additionally, using dynamic light scattering (DLS) experiments, we determined the conditions under which Pgk does not form aggregates were determined. The DLS experiments were performed on the Zetasizer Nano-S (Malvern Instruments version 7.12), which determines the molecular size and has a temperature controller, employs a 4 MW (megawatts), 633 nm semiconductor laser light source, and NIBS technology (Malvern Instruments, Ltd., UK). During the experiments, the temperature was adjusted via a Peltier unit. Data was analyzed using nano Zetasizer S DTS software (Malvern Instruments, Ltd., UK). The methodology consisted of filtering each protein buffer solution through a top filter with a 0.02 μm mesh aperture (Whatman GmbH) to remove foreign particles. Fifty microliters of the solution was introduced into a 3.0 mm quartz cell. The hydrodynamic diameter *d*(*H*) of both Pgk was analyzed over a range of 4–45 °C, with an interval of 0.5 °C, and with three replicates.

### 2.7 Measurement of the Enzymatic Activity of the Recombinant Pgk Protein

Pgk activity was determined by measuring the disappearance of reduced nicotinamide adenine dinucleotide (NADH) at 340 nm. The reaction mixture contained pure recombinant Pgk enzyme to which was added a solution of GAPDH (0.5 U/mL), NAD^+^ (0.1 mM) in buffer (HEPES 50 mM, KCl 100 mM, MgCl_2_10 mM, EDTA 1 mM, EGTA 1 mM, glycerol 10% (v/v), and Triton X-100 0.1% (v/v)). A 96-well plate (Corning Costar) was prepared by dispensing 1 μg of pure protein, increasing concentrations of 3-phosphoglycerate (disodium salt) and ADP into each well, and the volume was topped up to 200 μL per well. The mixture was incubated at 40 °C for 20 min, and the formation of NADH, a product of the coupled reaction, is determined by measuring the absorbance at 340 nm in the UV–visible Epoch spectrophotometer, BioTek Instruments, Inc. supported by the Gen5 All-In-One Microplate Reader software. Using a calibration curve performed with different concentrations of NADH, the concentration of NADH produced was determined, considering the molar extinction coefficient of NADH (6220 M^–1^ cm^–1^ at 340 nm) and 0.5 cm as the cuvette length. The procedure described previously was carried out to evaluate the effect of the compounds on enzymatic activity, adding a concentration of 5 μM of each compound to be assessed. Each experiment was performed in triplicate, and statistical analysis of the data was performed with GraphPad Prism 9 software.

### 2.8 Small-Angle X-ray Scattering (SAXS) Experiments

Small-angle X-ray scattering (SAXS) data for *C. glabrata* PgK was collected on the B21 beamline at the Diamond Light Source (Didcot, UK).^46^ The experiments were conducted in 20 mM Tris and 100 mM NaCl, pH 8.0, using an EigerX 4 M detector at a sample–detector distance of 3.7 m and a wavelength of 0.95 Å. In-line size-exclusion chromatography (SEC-SAXS) was employed for the construct. A 35 μL sample was injected at a flow rate of 0.075 mL/min onto a GE Superdex 200 increase 3.2/300 column at 15 °C. Scattering data were collected over 620 successive 3 s frames.

Data processing involved normalizing the transmitted beam’s intensity, radial averaging, and subtracting solvent-blank scattering. ScÅtter software (http://www.bioisis.net/) was used to identify and select frames corresponding to sample scattering and the appropriate solvent blank. Subtracted frames were scaled and averaged to produce the final SAXS profile.

The initial analysis of the SAXS-derived parameters for all the SAXS experiments was performed in PRIMUS, which is included in ATSAS suite.^47^ The results are given in Table 1.

**Table 1:** SAXS Sample Details, Data Collection, Analysis, and 3D Modeling Details^a^

sample	Pgk
organism	*C. glabrata*
Data collection parameters	
synchrotron beamline	B21/Diamond Light Source (Harwell, UK)
detector (distance mm)	EigerX 4M-Detrics (3685.6)
beam size (mm)	0.05 × 0.05
energy (keV)	13.1
*q* range (A^–1^)	0.0045–0.34
exposure time (s)	3.0
number of frames	620
temperature (K)	287
mode	SEC online: Superdex 200 3.2/300 inc
Structural parameters	
injection concentration (mg/mL)	5.0
*q* interval for Fourier inversion (Å^–1^)	0.009–0.240
Rg [from *P*(*r*)] (Å)	28.90 ± 0.07
Rg [from Guiner approximation] (Å)	28.88 ± 0.07
sRg limits [from Guiner approximation]	0.26–1.29
*D*_max_ (Å)	103
porod volume estimate (nm3)	79
molecular mass (kDa)	
estimated (probability)	47 (91%)
from sequence	45
ambiguity score	1.93 (3D reconstruction might be ambiguous)
Software employed	
primary data reduction	DAWN pipeline (Diamond Light Source, UK)
data processing	ScÅtter IV, ATSAS 4.0
modeling	GASBOR
computation of model intensities	CRYSOL
SASBDB code	SASDXB2

a *q* = 4pi × sin­(θ/λ).

The low-resolution SAXS envelope for profilin was generated using the GASBOR program.^48^ Model fitting to experimental data was performed using the CRYSOL program,^49^ and further refinement was achieved through normal-mode analysis (NMA) using the SREFLEX^50^ algorithm, all the programs included in the ATSAS suite.

The SAXS data and models have been deposited in the Small Angle Scattering Biological Data Bank (SASBDB) and are available under accession code SASDXB2.

### 2.9 Bioinformatics Analysis

#### 2.9.1 Sequence Retrieval and Selection

Protein sequences of phosphoglycerate kinase (Pgk) from *C. albicans*, *C. glabrata*, *T. cruzi,* and *Homo sapiens* were retrieved in the FASTA format from the nonredudant protein database (NCBI). Accession numbers were as follows: CaPgk: XP_711323.1, CgPgk: UCS22915.1, TcPgk: EAN97117.1 and HsPgk: NP_000282.1. To ensure sequence representativeness, entries were selected based on completeness, annotation quality, and sequence length. Redundant entries, partial isoforms, and uncharacterized variants were excluded.

#### 2.9.2 Domain Identification

Functional domain analysis was performed using the InterPro server (https://www.ebi.ac.uk/interpro/). Each sequence was submitted using the default parameters. InterProScan integrates multiple databases, including Pfam and SMART, to identify conserved protein domains. Identified domains were recorded and compared across species.

#### 2.9.3 3D Protein Structure Modeling

The monomeric 3D structure of *C. glabrata* Pgk was predicted using AlphaFold3. The corresponding FASTA sequences were uploaded, and the structure was predicted using the default settings. AlphaFold3 was used to predict the structure, including cofactor binding, by leveraging its advanced integrated cofactor modeling capabilities.

#### 2.9.4 Structural Quality Assessment

The predicted models were evaluated using SAVES v6.0 (https://saves.mbi.ucla.edu), which integrates the following tools: ERRAT v2.0: overall quality factor; Verify-3D v1.0:3D/1D profile compatibility; and PROCHECK v.3.5.4: Ramachandran plot analysis. The structural quality analysis was performed by considering thresholds and manually inspecting Ramachandran plots. The model with the best scores was selected for further analysis.

#### 2.9.5 Energy Minimization

The model was submitted to the YASARA Energy Minimization Server to improve intramolecular interactions. Energy minimization was performed using the AMBER14 force field with default parameters (5000 steps until convergence). After minimization, the model was evaluated by using the Score function in YASARA to confirm energy minimization.

#### 2.9.6 Modeling of the Ligands

The chemical structures of the antifungals were obtained from the PubChem database (accessed March 2025), and their identification codes are Amphotericin B: 5280965, Fluconazole: 3365, and Nystatin: 6433272. The 2D structures were downloaded in the SDF format to be imported into Avogadro, an open-source molecular editor selected for its user-friendly interface and integrated tools for 3D structure generation and energy minimization. This software automatically generated three-dimensional conformations and subjected to energy minimization using the MMFF94 force field.^51^ This force field was selected due to its proven accuracy and parametrization for various pharmacologically relevant organic molecules. The minimization was carried out using the steepest descent algorithm, with no positional constraints, a maximum of 500 steps and a default convergence criterion of approximately 1 × 10^–7^ kcal/mol for the energy change between steps. The minimized structures were then exported in the PDB format for molecular docking studies.

### 2.10 Molecular Docking

Molecular docking studies were conducted by using AutoDock Vina 1.1.2 through the PyRx 0.8 interface. Protein and ligand structures were initially imported in the PDB format and converted to the PDBQT format within PyRx. Proteins were defined as rigid macromolecules, while ligands were treated as flexible with rotatable bonds automatically assigned by the software.

#### 2.10.1 Protein Preparation

Protein model of Pgk from *C. glabrata* was prepared using YASARA. Water molecules and nonessential heteroatoms were removed. Polar hydrogens and partial atomic charges were added. Protein torsions were kept rigid, consistent with the rigid receptor model employed by AutoDock Vina. The protein was placed in an explicit aqueous environment to validate structural integrity, but docking was performed under vacuum conditions within PyRx.

#### 2.10.2 Ligand Preparation

Ligands (amphotericin B, fluconazole, and nystatin) were previously energy minimized in Avogadro using the MMFF94 force field. In PyRx, torsional flexibility was automatically assigned to all rotatable bonds, protonation states were kept at physiological pH (7.4), and Gasteiger charges were assigned by default during the PDBQT conversion.

#### 2.10.3 Docking Parameters

A blind docking approach allowed an unbiased exploration of the entire receptor surface. The exhaustiveness parameter was set to 10. Autodock Vina uses a stochastic global optimization method (iterated local search) to identify the most favorable ligand–receptor binding poses based on predicted binding free energy.

For Pgk:

Grid center: X:-0.2037, Y:0.8441, Z:0.5702.

Grid size: X: 60.6525 Å, Y: 55.8401 Å, Z: 69.0852 Å.

The best binding poses were selected based on the lowest binding free energy values reported by AutoDock Vina.

#### 2.10.4 Postdocking Analysis

Docked complexes were visualized using UCFS Chimera X for three-dimensional (3D) analysis and BIOVIA Discovery Studio Visualizer 2024 for two-dimensional (2D) interaction mapping. Interaction types, such as hydrogen bonds, hydrophobic interactions π, systems, and electrostatic interactions were identified to assess ligand–protein binding quality.

### 2.11 Prediction of Toxicological Properties

The pkCSM server was used to predict the toxicity the compounds evaluated could present when administered as drugs in humans.^52^ In the server interface (https://biosig.lab.uq.edu.au/pkcsm/), the structures of each compound were loaded and the default parameters were followed.

## 3 Results and Discussion

### 3.1 Evaluation of the Amino Acid Sequence of the Pgk Protein from *C. glabrata*

The amino acid sequence of the Pgk protein of *C. glabrata* was analyzed with InterPro. This software uses databases to identify the regions and domains that comprise the protein. Pgk from *C. glabrata* showed, as has been identified in orthologs of this protein from other organisms, that it corresponds to a multidomain protein, where approximately the first 113 amino acids in the N-terminal region correspond to a domain called PAS (Per-ARNT-Sim), which is formed by folded beta-sheets, alpha-helices, and loops, as well as a PGK site and the conserved site (amino acids 18–28) formed by a folded beta-sheet and a loop (Figure 1). Results in agreement with orthologous proteins in other organisms such as *T. cruzi*.^30^

**Figure 1:**
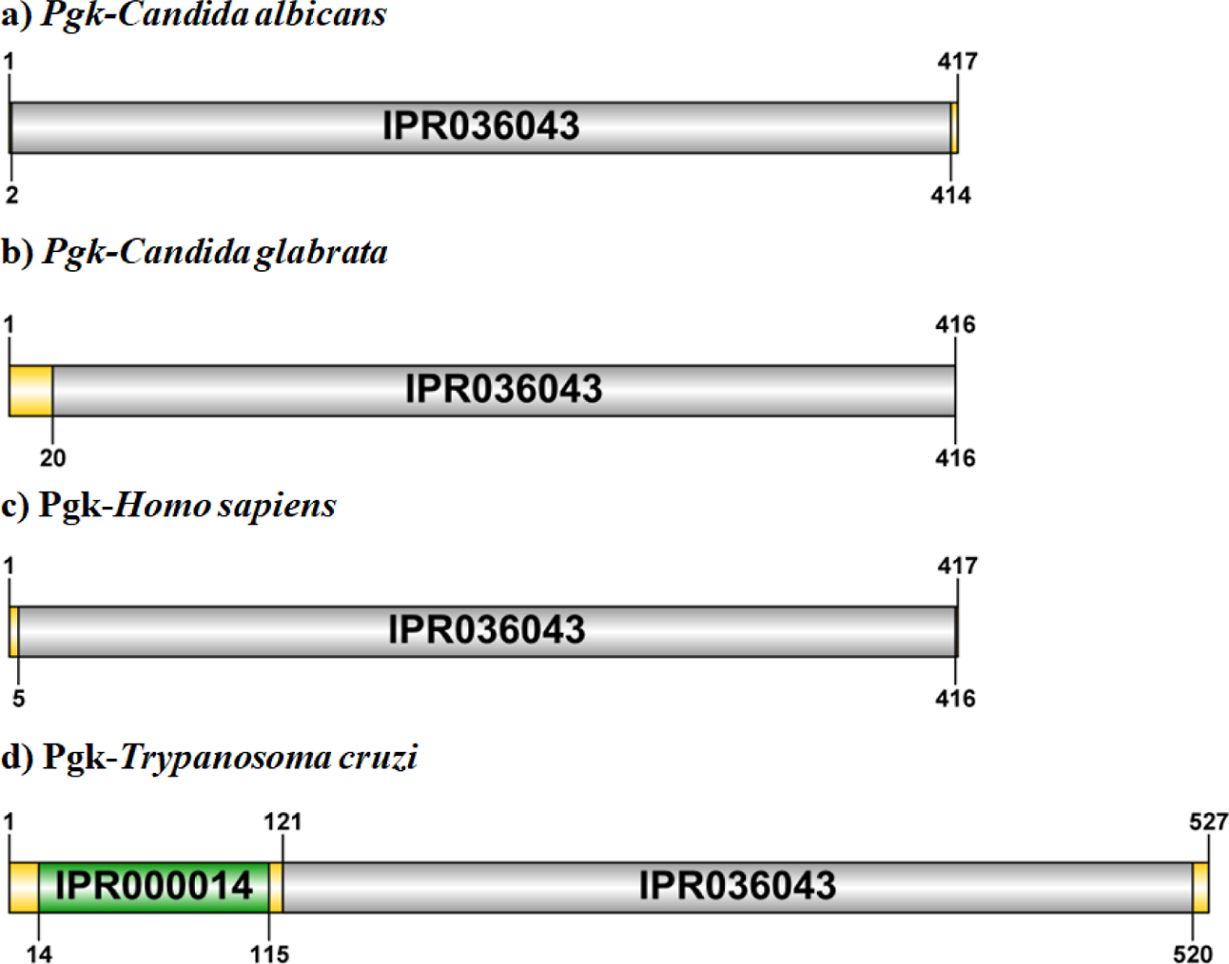
Predicted domains in the amino acid sequence of Pgk from C. glabrata compared to Pgk from other organisms: a) *C. albicans*; b) *C. glabrata*; c) *Homo sapiens*; d) *Trypanosoma cruzi*.

### 3.2 *In silico* 3D Modeling of the Pgk Protein

The function of a protein depends on its 3D structure; therefore, determining the structure of a protein both in silico and by any of the experimental methods is an arduous and intricate task.^53–55^ The difficulties are mainly due to the discrepancy between amino acid residue sequences and the number of experimentally solved 3D protein structures.^56^ However, beyond how complicated 3D structure resolution can be, it is of special emphasis to have the 3D structure of proteins and enzymes with therapeutic targeting possibilities. This is because structure-based drug design starts with the choice of target protein. In the case of Pgk from *C. glabrata*, it is an immunodominant protein, which makes it a potential therapeutic target against IC.^21–24^ Currently, the 3D structure of this protein is not available, which is why, as a first step before designing any drug against Pgk, we performed in silico modeling using the AlphaFold3 and I-TASSER platforms.^57,58^ The 3D model of the complete *C. glabrata* Pgk sequence showed that the PAS domain is located in the N-terminal region of the protein, the PGK catalytic domain is located between the PAS domain and the C-terminal region (Figure 2). The protein model shows that it consists of 23 helices and 16 beta sheets (Figure 2).

**Figure 2:**
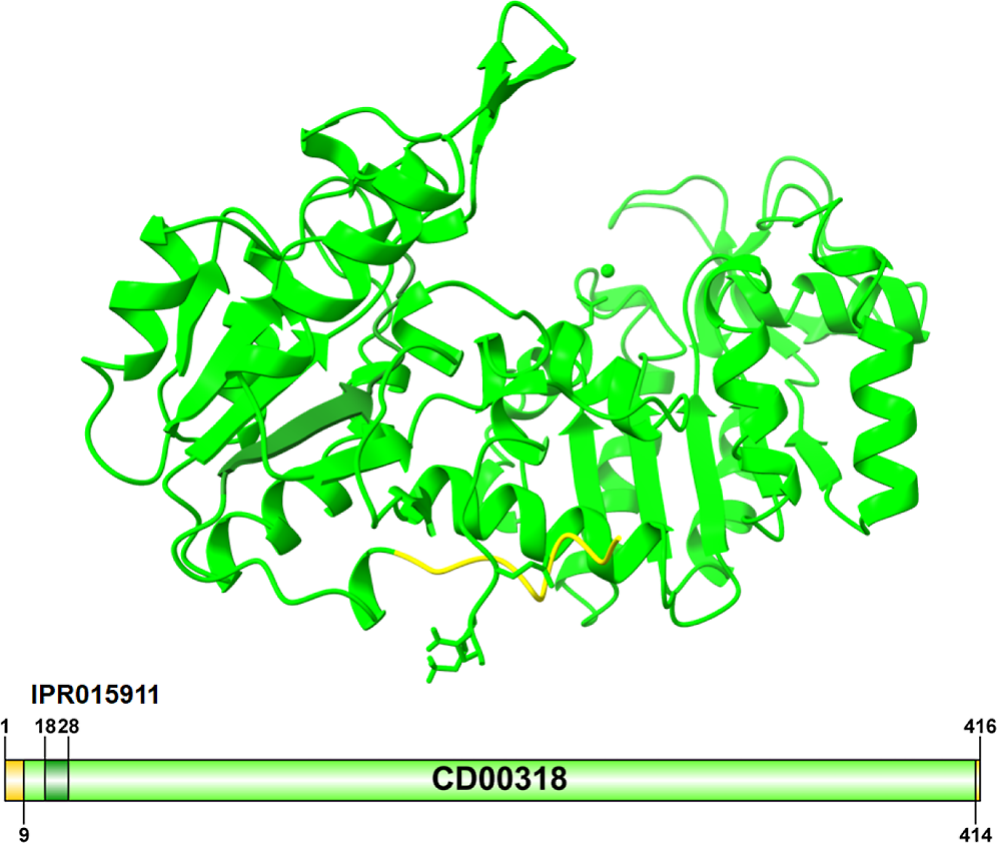
3D model of the Pgk monomer from *C. glabrata*. Illustration of the domains that make up the protein using InterPro. The 3D structure was modeled by AlphaFold 3.

Of the domains that make up Pgk, the PAS domain is relevant because it has been found in all five kingdoms of life, in organisms, such as archaea, eubacteria, cyanobacteria, fungi, plants, insects, and mammals.^58,59^ Among the functions of proteins possessing this domain are those that act as transcription activators, multidomain sensors, transcription factors, ligand binding, and cofactors.^59^ These domains adopt a folding that some authors have called a glove-like folding, which is constituted by two beta sheets and three alpha helices, a connector formed by an α helix diagonally crossing the beta sheets, and a beta scaffold formed by three antiparallel beta sheets.^30,60–62^ As shown in Figure 3, even though a similar region is observed in the N-terminal region at the alpha helices and beta-sheet positions, the difference in the number of these is notable. Specifically, the PAS domain of Pgk-*C. glabrata* consists of 8 alpha helices (Figure 3B) and 5 beta sheets (4 parallel and 1 parallel) (Figure 3C).

**Figure 3:**
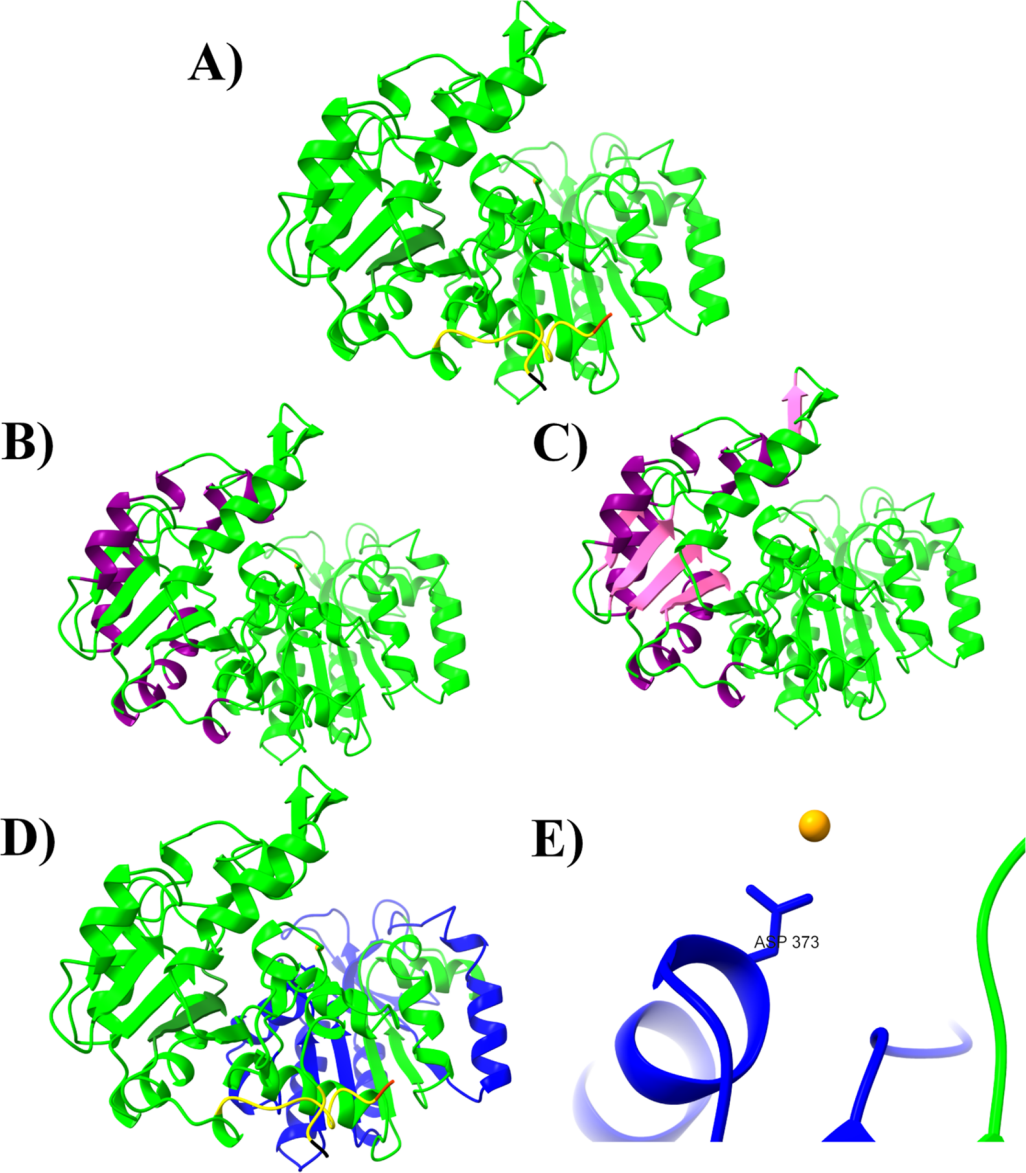
3D structure domains of Pgk from *C. glabrata*. (A) Pgk-*C. glabrata*. Red: amino end; Black: carboxyl end. (B) PAS domain (amino acids 1 to 133), formed by 8 alpha-helices. Conformed by amino acids: 9–11, 38–43, 45–53, 73–75, 78–88, 101–108, 121–123, and 125–128. (C) PAS domain: 5 beta-strands. Conformed by the amino acids: 18–22, 57–61, 92–95, 114–118 (Parallel), and 130–133 (Antiparallel). (D) Blue active site: amino acids 253–390. (E) Active site (blue) and interaction of residue ASP373 with Mg^2+^ (1.248 Å).

The similarity in this region of *C. glabrata* Pgk with respect to other Pgk suggests that its function would be similar to these, one of these being the binding to cofactors and ligands.^63^ This is an essential function for Pgk from *C. glabrata* to be considered a therapeutic target. Meanwhile, the divergence in the number of alpha helices and beta sheets of *C. glabrata* Pgk concerning other orthologous proteins from different organisms could be attributed to sequence evolution between species. The catalytic site of Pgk structures from other organisms solved by X-ray crystallography is conserved.^64^ In the 3D structure of Pgk-*C. glabrata*, the active site is indicated (Figure 3D,E). The N-terminal region contains the 3-phosphoglycerate-1,3-bisphosphoglycerate binding site and the C-terminal region contains the nucleotide binding site (ADP/ATP).^65^ These regions were also found in the predicted structure of *C. glabrata* Pgk (Figures 2 and 3). Validation was performed to assess the quality of the generated 3D model using an external SAVES server. This is because, although AlphaFold can predict disordered regions, it has problems when modeling loops or folds,^66^ which is why ModLoop was used, a server that allows modeling these specific segments by using the loop modeling routine in MODELER, predicting conformations by satisfying spatial constraints based on energy stabilization, without relying on a database of known structures,^67^ and since only the atomic coordinates of the loop are intervened, it is guaranteed that there is no alteration of other regions of the structure modeled in AlphaFold. In addition, using the Yet Another Scientific Artificial Reality Application, YASARA, it was possible to energetically minimize the structure, which means that it will be stable and in a more consistent conformation with the one it would have in vitro or in vivo.

Furthermore, after going through these optimizations, the proteins were re-evaluated in the SAVES server to ensure their suitability, through essential indicators such as ERRAT, which evaluates the overall quality of the structure, detecting possible errors in the geometry, such as incorrect bond angles, anomalous atomic distances, or improper rotations in the atoms. A score above 90% indicates a high probability that the structure is reasonable and free of apparent defects. Verify-3D evaluates the quality of the model by verifying that the atoms in the three-dimensional structure are physically plausible when compared to residue profiles in known structures (solved by X-ray crystallography), with a score above 60% indicating a good prediction of atom positions and expectations based on experimental structures, reflecting that the model structure has not only a valid geometry but also a high probability of being in a biologically viable conformation. Procheck evaluates the conformation of individual residues in the model based on assessing the phi-psi angles of the residues in the structure and comparing them to known conformations. Relying on Ramachandran plots, the distribution of these angles in the highly stable and common FR favored regions, the less common AR allowed region, the rare but possible GR generous regions, and the DR unfavorable regions. If a residue is in unfavorable areas, then it indicates that its conformation is not biologically plausible or is too far away from the preferred configurations for that type of residue. The data from the quality assessment of Pgk were favorable, as shown in the Ramachandran plot (Figure S1). After performing the 3D model evaluation, we ensured that the obtained model is reliable and can be used to perform molecular docking experiments with the selected molecules. Once the 3D model of Pgk from *C. glabrata* was validated, we proceeded with the expression and purification of the protein to obtain the 3D structure using the SAXS crystallographic technique. These results will allow the possible design of drugs against this protein.

### 3.3 Expression and Purification of the Recombinant Pgk Protein

*E. coli* BL21 (DE3) cells were transformed with plasmid pET19b-PGK-Cg, and protein expression was induced. Once the protein was expressed, it was purified by Ni­(II) ion affinity chromatography. The purity of Pgk was corroborated by SDS-PAGE (Figure S2). The results showed that the concentration and purity of the *C. glabrata* Pgk protein were adequate for enzyme activity determination and 3D structure determination by SAXS.

### 3.4 Dynamic Light Scattering Characterization

Pure Pgk from *C. glabrata* was evaluated by dynamic light scattering (DLS), where aggregate formation was detected, and the best conditions were determined, where the expected hydrodynamic radius (Rh) for these proteins was obtained. A possible hydrodynamic radius of 5.1 nm was determined. The DLS analysis reports the particle size and volume occupied by the particle in the sample. It is then deduced that the particle occupying the most significant volume is the most abundant in the sample. In each DLS measurement, the variation of the size (*d*·nm) concerning the volume (%) of the protein was analyzed. From the experimental data that showed the values of hydrodynamic diameter (*d*_h_) from the maxim peak, the condition that not only presents the average *d*_h_ closest to the expected one but also the one that when plotting its size (*d*·nm) concerning the volume in the percentage indicates that the particle representing the Pgk is the most abundant in the sample, was selected.

Thus, the conditions that showed the closest to expected *d*(*H*) values were at pH 7.4, 21 °C, and *d*(*H*) of 11.08 nm (Figure 4). As seen in Figure 4, the particle representing Pgk is the most abundant in the sample. Based on this result, we proceeded with structural determination by SAXS.

**Figure 4:**
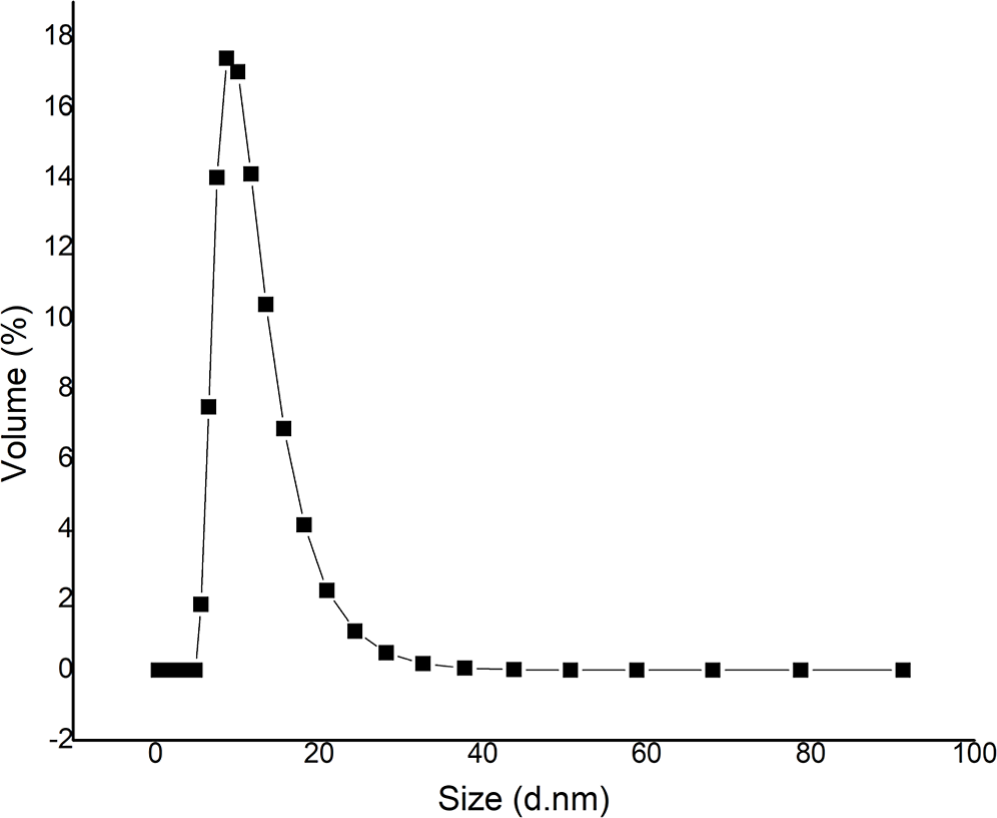
Variation of Pgk size (*d*, nm) versus volume (%) obtained by DLS at 21 °C, pH 7.4.

### 3.5 Structural Analysis by Small-Angle X-ray Scattering (SAXS)

For PgK, data acquisition utilized was size-exclusion chromatography coupled with SAXS (SEC-SAXS), allowing for purification, followed by the SAXS measurement (Figure 5). The SEC-SAXS elution profile displayed mostly a single prominent peak, indicating a well-defined homogeneous monomeric state.

**Figure 5:**
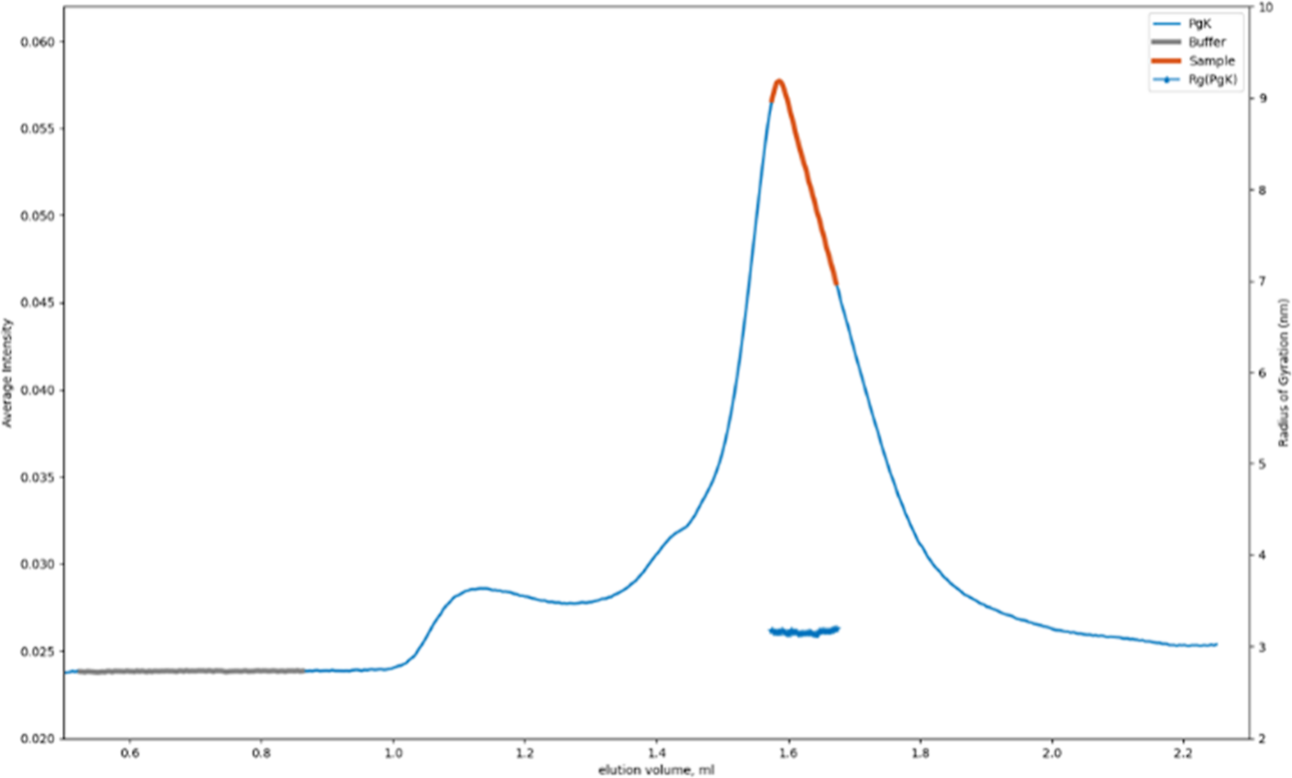
SEC-SAXS data as SAXS averaged intensity vs elution volume. The selected frames for the sample and buffer are gray and red, respectively. From the subtracted sample-buffer frames, the estimation of the radius of gyration was shown, which is consistent across the frames across the elution peak.

The radii of gyration (*R*_g_) values, calculated from individual SAXS frames across the elution peak, were consistent: 2.9 ± 0.1 nm. These frames were scaled, averaged, and subsequently used for further analysis. SAXS parameters are displayed in Table 1, while Figure 6 summarizes the SAXS experimental data and their principal data analysis. The one-dimensional (SAXS) experimental data (Figure 6a) were evaluated by using the Guinier plot (Figure 6 b), indicating good data quality. The estimated molecular weight of 47 kDa closely matched the value calculated from the sequence (Table 1). The dimensionless Kratky plot (Figure 6c) and the pair distribution function (Figure 6d) suggested that PgK possesses a mostly compact structure with some inherent flexibility. This interpretation was further supported by a low-resolution Cα model generated using the GASBOR program (Figure 7a).^48^ This low-resolution model accommodated the predicted model from AlphaFold (Figure 7b) and that after minimization. The model’s agreement with the experimental SAXS data was validated by fitting the calculated SAXS curve generated with CRYSOL to the experimental data (Figure 8).^49^ In conclusion, the SAXS experiment was very useful not only to confirm the good condition of the protein in the buffer but also to have experimental full agreement with the prediction.

**Figure 6:**
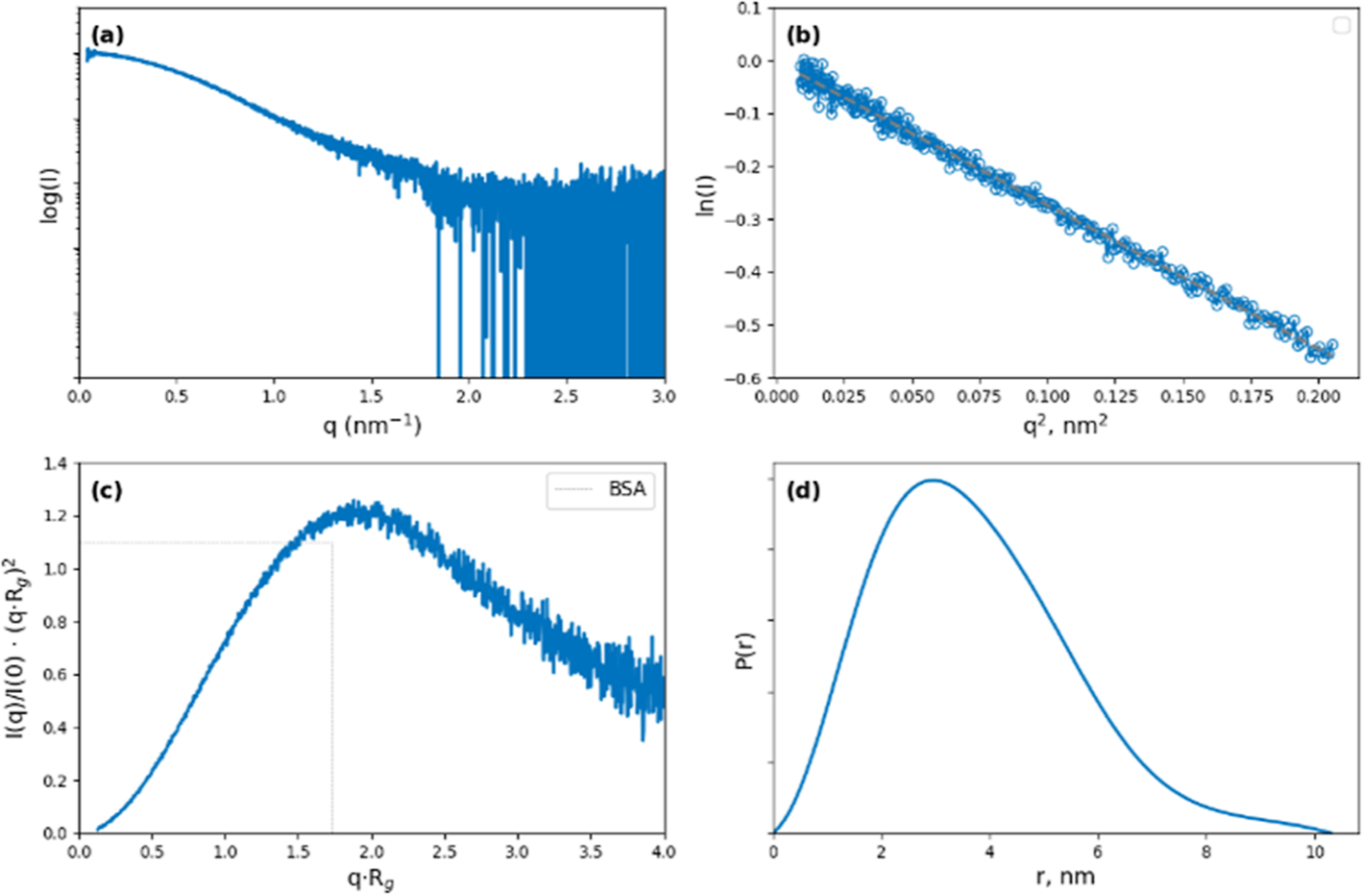
(a) Experimental SAXS; (b) Guiner region and the linear fitting from which the *R*_g_ is estimated; and (c) dimensionless Kratky plot. The intersection of the dotted black trace corresponds to the value of a bovine serum albumin (a globular protein used as a standard on SAXS experiments); (d) pair-distribution function (Pr) from which the maximum size (*D*_max_) was estimated.

**Figure 7:**
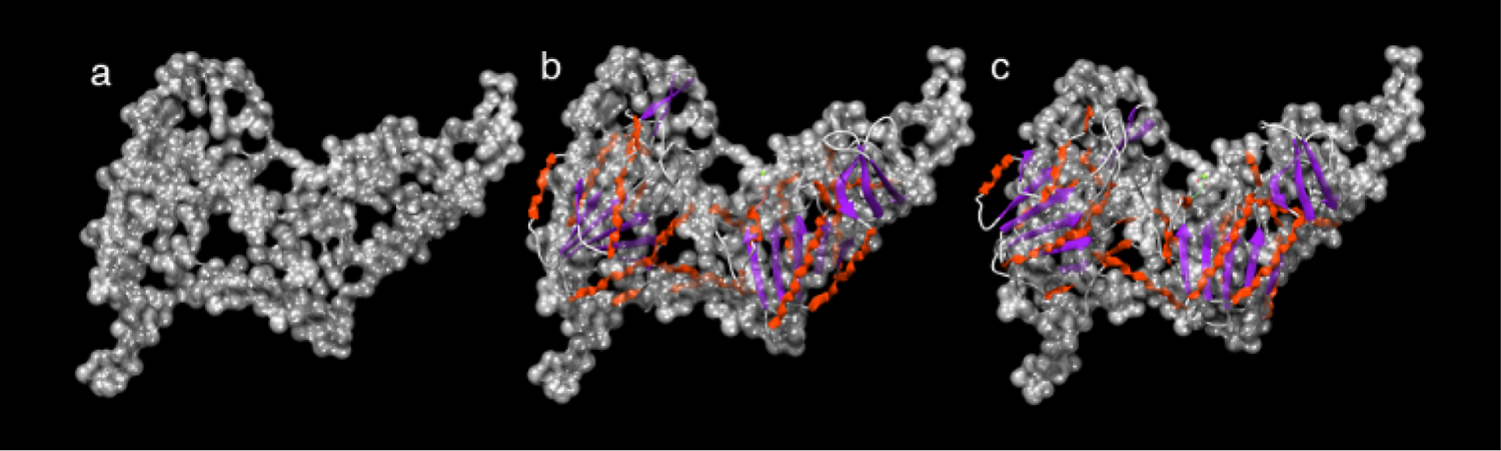
(a) SAXS Cα model generated using GASBOR; (b) AlphaFold2-predicted model; and (c) energy-minimized structure (YASARA), colored by secondary structure elements, each fitted to the GASBOR Cα model shown in gray.

**Figure 8:**
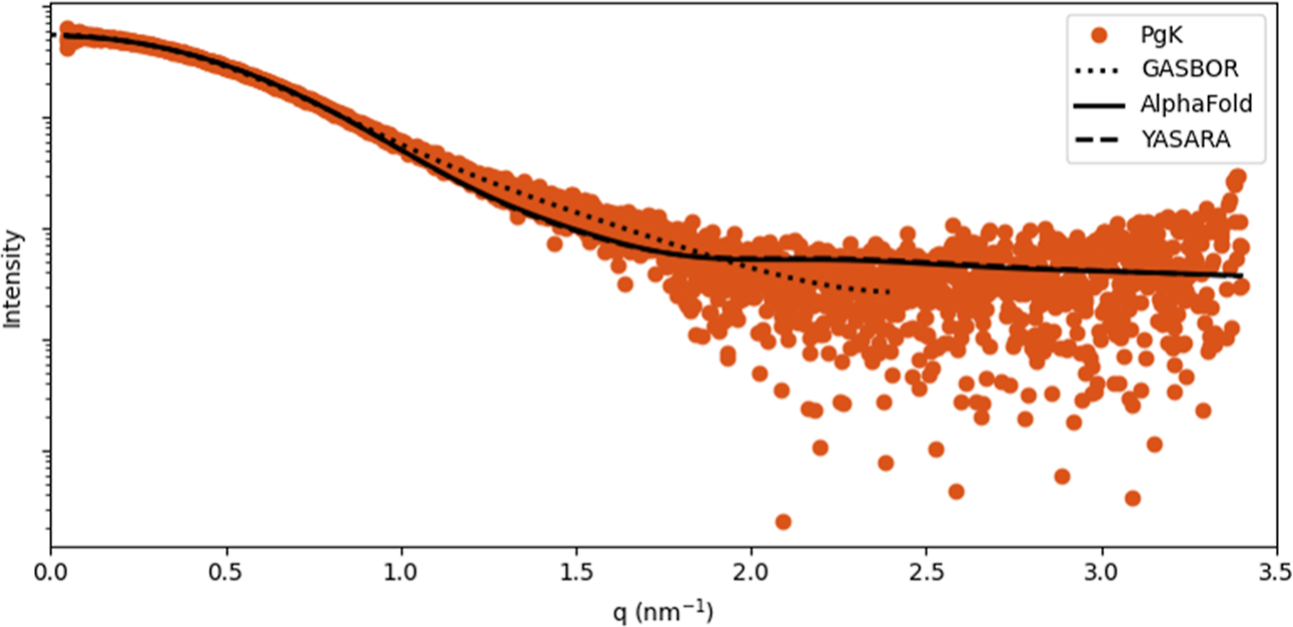
Comparison of SAXS Cα model generated by GASBOR with AlphaFold2-predicted and YASARA energy-minimized structures, fitted to the experimental scattering data.

The Kratky plot (Figure 6) shows the conformational state of the protein, nonaggregated state.

A comparison of our SAXS-derived Rg and Dmax with the predicted AlphaFold model (Figure 8) represents the fitting of calculated model with the experimental data.

### 3.6 Molecular Docking and Evaluation of Pgk as a Potential Therapeutic Target against IC

Accurately determining the structure of target proteins is critical in the structure-based drug design. Thus, based on the 3D structure of Pgk from *C. glabrata* solved by SAXS, we set out to perform molecular docking and evaluation of this protein as a potential target against IC, which will allow us to obtain essential information on its functional mechanisms, binding sites, and interactions with potential drug candidates. To determine the possible interaction between Pgk and drugs routinely used against IC, we chose three antifungals of different classes: amphotericin B, nystatin, and fluconazole. In addition, two compounds originally developed for other therapeutic purposes, nilotinib and netupitant, were included based on previous studies that reported theoretical interactions between them and Pgk from *C. albicans* and *C. glabrata*,^68^ suggesting they may have some therapeutic effect on this enzyme. Using the PyRx program, molecular docking simulations were performed in AutoDock Vina to predict the affinity energy value between Pgk and each of the selected compounds. A two-way analysis of variance (ANOVA) performed on the data obtained from molecular docking indicates that the affinity energy depends on the protein and the compound with which it is docked. The data found shows a significant difference (*p* value < 0.0001) in the predicted affinity energy obtained. The average affinity energies (kcal/mol) are shown in Table 2. As shown in the table, amphotericin B and nystatin exhibit similar affinity values (−7.3 and −7.4 kcal/mol, respectively), whereas fluconazole showed a weaker predicted interaction (−5.8 kcal/mol). Nilotinib and netupitant showed predicted affinities of −8.3 and −6.8 kcal/mol, respectively, with nilotinib exhibiting the highest binding affinity for Pgk among all tested molecules.

**Table 2:** Affinity Energy (in kcal/mol) of the Different Compounds Evaluated and Phosphoglycerate Kinase (Pgk) from *C. glabrata*

ligand	Pgk
amphotericin B	–7.3
nystatin	–7.4
fluconazole	–5.8
nilotinib	–8.3
netupitant	–6.8

The results of molecular weight estimation via Bayesian inference (e.g., DATMW) showed that the estimated molecular weight for Pgk is 47 kDa and by sequence 45 kDa. These data together confirm the monomeric state (Table 1).

Data show that Pgk-*C. glabrata* possibly does interact with these antifungals. In order to know the amino acids with which the protein possibly interacts; molecular docking was performed. The docking results provided additional structural insights into the possible binding interactions between Pgk from *C. glabrata* and the new compounds. Although these findings are based on theoretical models, they allowed us to hypothesize which amino acid residues could participate in potential ligand–protein interactions. In the case of the Pgk-Amphotericine B complex, the interactions are determined by carbon–hydrogen bonds of Glu-342 at 3.37 Å with the C of the antifungal, a metal-acceptor interaction of Mg with the O of the drug at 2.58 Å, and three hydrogen bond-type interactions of residues Asp373 at 2.07, Arg66 at 2.59 Å with the O from C=O of the antifungal, and Arg122 at 2.34 Å from the O of the drug (Table 3, Figure 5).

**Table 3:** Links between Pgk-*C. glabrata* and Antifungals

receiver	ligand	atom from ligand	amino acid	kind of bond	distance (Å)
Pgk-*C. glabrata*	Amp B	C	GLU 342	carbon–hydrogen bond	3.37
		O	Mg	metal-acceptor	2.58
		H from OH	ASP373	hydrogen bond	2.07
		O from C=O	ARG66	hydrogen bond	2.59
		O	ARG122	hydrogen bond	2.34
	nystatin	H from NH	VAL99	hydrogen bond	2.23
		H from OH	ASP97	hydrogen bond	2.13
		O from OH	HIS124	carbon–hydrogen bond	2.48
		H from OH	GLU126	hydrogen bond	2.38
		H from OH	GLU126	hydrogen bond	2.42
		H from OH	ASP144	hydrogen bond	2.71
	fluconazole	fluorine	ALA213	hydrogen bond	2.51
		fluorine	GLY212	carbon–hydrogen bond	3.53
		fluorine	ASP373	halogen	3.31
		fluorine	PRO337	carbon–hydrogen bond	2.54
		benzene	PRO337	Pi-Alkyl	5.35
		benzene	VAL340	Pi-Alkyl	5.28
		H from triazole	PRO338	hydrogen bond	2.24
		H from triazole	LEU312	hydrogen bond	2.68
		C from triazole	GLY311	carbon–hydrogen bond	3.47
	nilotinib	H from NH	ILE387	hydrogen bond	2.46
		benzene	ALA42	Pi-alkyl	5.19
		benzene	PRO45	Pi-alkyl	4.89
		O from C=O	LEU187	carbon–hydrogen bond	2.49
		benzene	LYS190	Pi-alkyl	3.96
		F	THR189	carbon–hydrogen bond	2.6
		F	THR186	carbon–hydrogen bond	2.46
		CF3	PHE186	Pi-alkyl	4.84
		F	GLN193	hydrogen bond	2.46
	netupitant	F	PRO337	halogen (fluorine)	3.48
		F	GLY339	halogen (fluorine)	3.00
		F	GLY212	carbon–hydrogen bond	2.48
		CF3	ILE255	alkyl	5.12
		CF3	PHE290	Pi-alkyl	5.43
		CF3	LEU312	alkyl	5.08
		CF3	VAL340	alkyl	5.12
		benzene	VAL340	Pi-alkyl	5.15
		benzene	VAL340	Pi-alkyl	5.40
		benzene	GLU342	Pi-anion	3.70
		C	GLU342	carbon–hydrogen bond	3.69
		benzene	ALA213	Pi-alkyl	4.38

In the Pgk–Nystatin complex, the interactions identified were of the hydrogen bond type with Val99 at 2.23 Å, Asp97 at 2.13 Å, Glu126 at 2.38 and 2.42 Å, and Asp144 at 2.71 Å of H from OH of the nystatin of Pgk (Table 3, Figure 10). The His124 of Pgk was attached to the O from OH of the antifungal by carbon–hydrogen bonding (Table 3, Figure 10).

**Figure 9:**
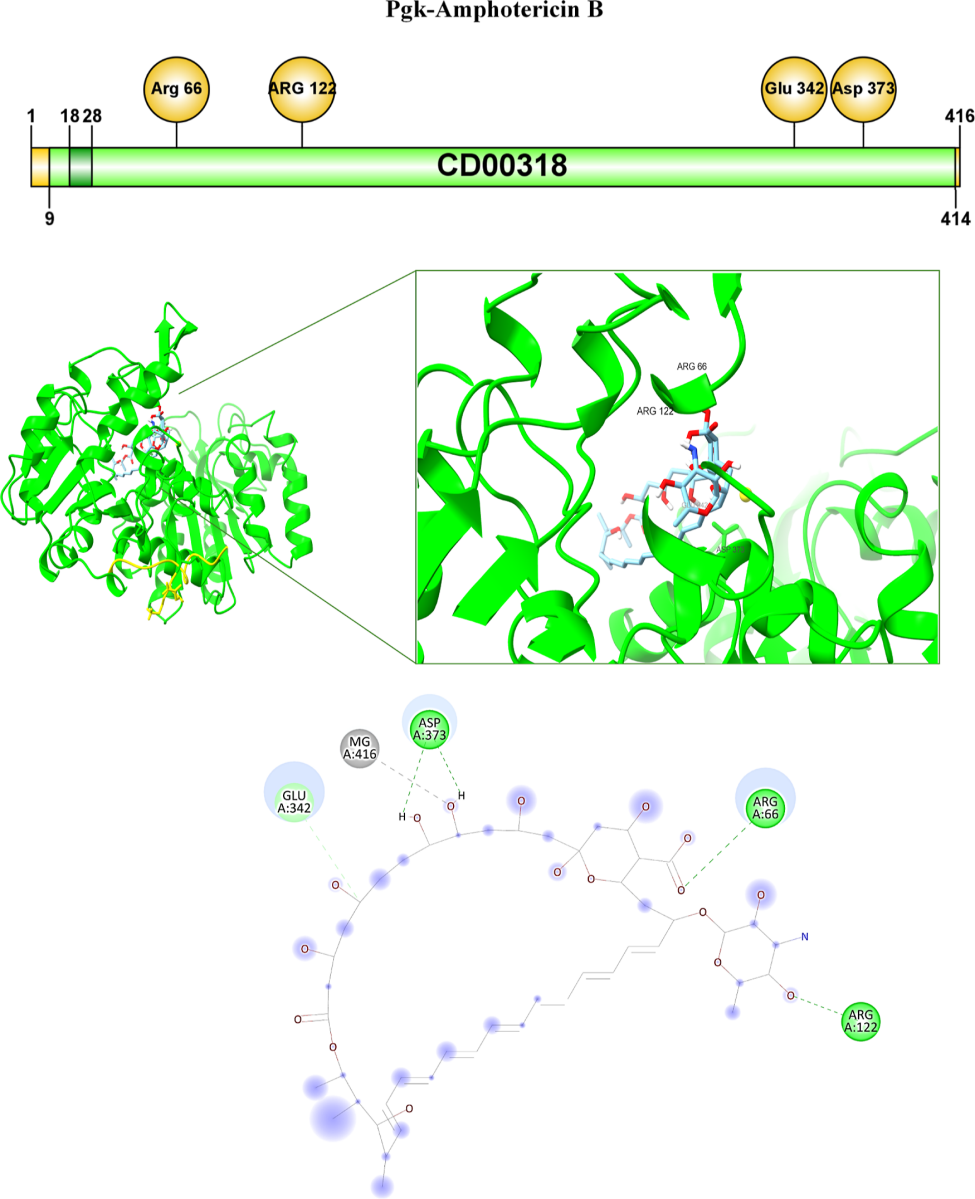
Interaction between Pgk of *C. glabrata*with amphotericin B. The binding of the Pgk-antifungal complex is shown with the interaction of specific amino acids.

**Figure 10:**
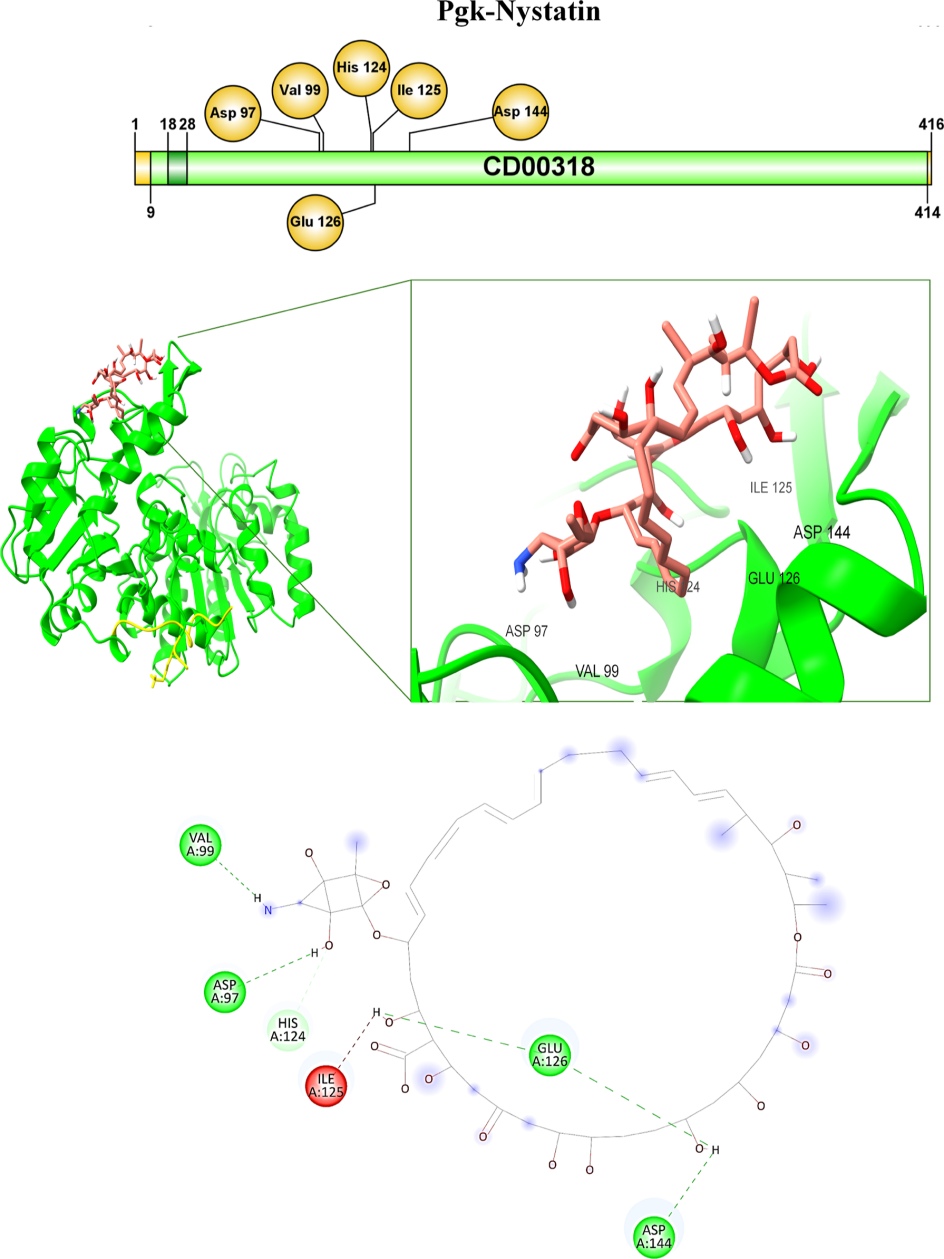
Interaction between Pgk from *C. glabrata*with the antifungal drug nystatin. The binding of the Pgk-ligand complex is shown with the interaction of specific amino acids.

While the amino acid residues involved in the interaction in the complex formed by Pgk–Fluconazole are Ala213 at 2.51 Å, Gly212 at 3.53 Å, Asp373 at 3.31 Å, and Pro337 at 2.54 Å from the fluorine group of the antifungal, Val340 at 5.28 Å and Pro338 at 5.35 Å from the benzene of the ligand, Leu312 and Gly311 with the H from OH of the antifungal (Table 3, Figure 11).

**Figure 11:**
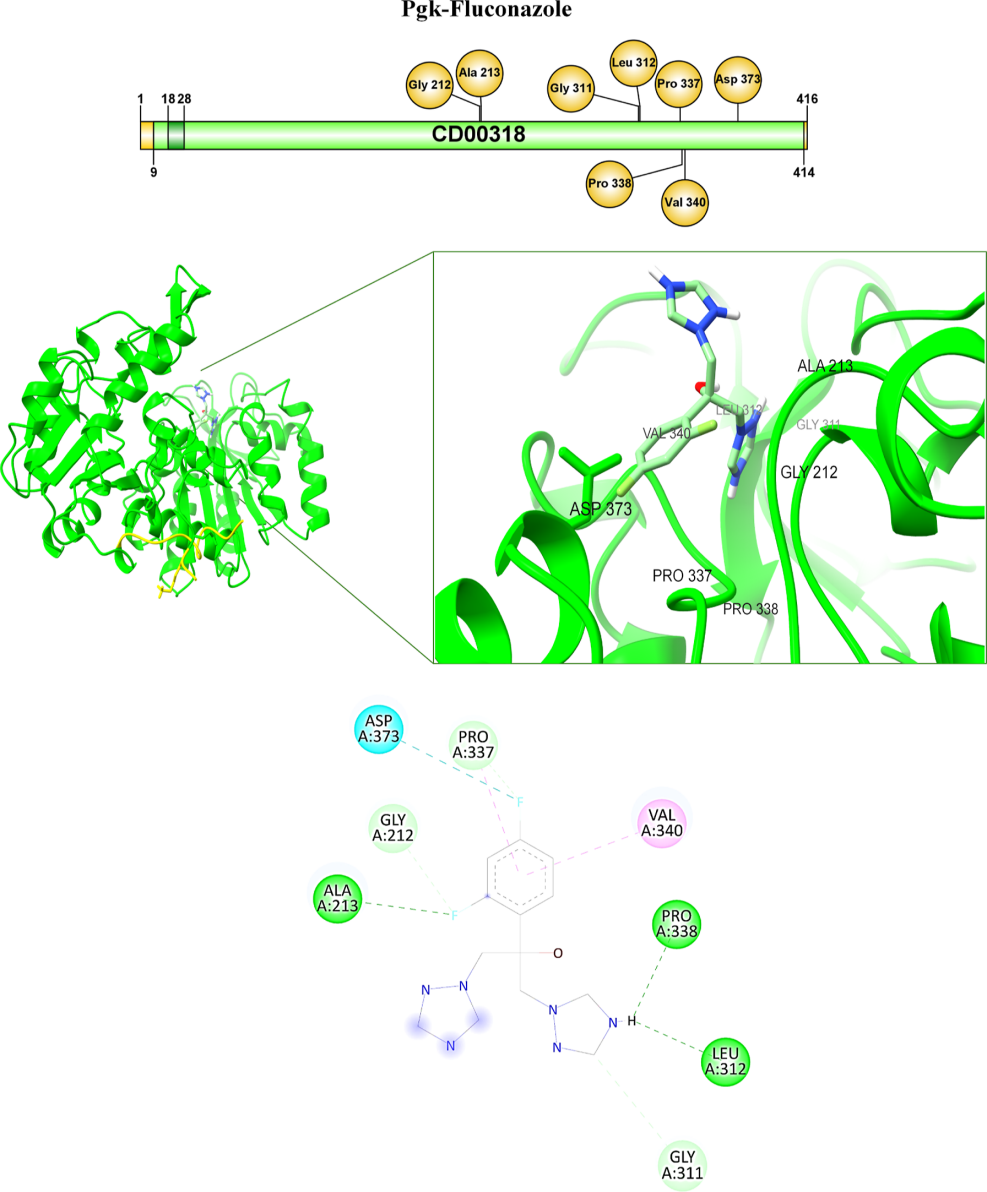
Pgk-Fluconazole interaction. Binding in the Pgk-drug complex is shown with the interaction of specific amino acids.

In the complex formed by Pgk–Nilotinib, the interacting residues include Ile387 at 2.46 Å with the H form the NH of the ligand, Leu187 at 2.49 Å and Thr189 at 2.6 Å with the O and F atoms, respectively, through carbon–hydrogen bonds, and Gln193 at 2.46 Å with the F via hydrogen bonding. Pi-alkyl interactions were identified between the benzene ring of nilotinib and Ala42 at 5.19 Å, Pro45 at 4.89 Å, Lys190 at 3.96 Å, and between the CF_3_ group and Phe186 at 4.84 Å (Table 3, Figure 12).

**Figure 12:**
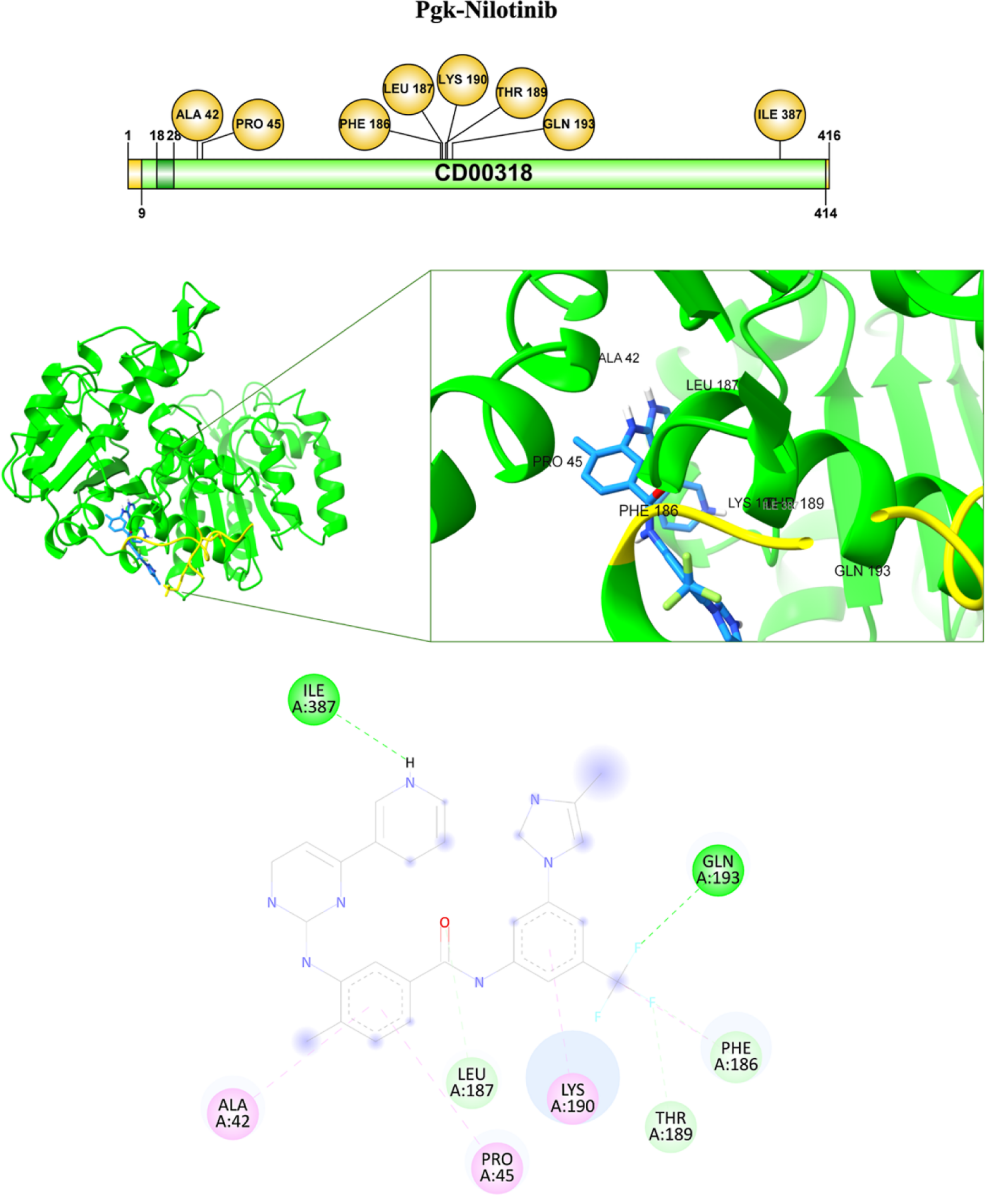
Pgk–Nilotinib interaction. The binding of the Pgk-ligand complex is shown with the interaction of specific amino acids.

In the Pgk–Netupitant complex, the fluorine atom of the ligand interacts with Pro337 at 3.48 Å and Gly339 at 3.00 Å through halogen bonds and with Gly212 at 2.48 Å via a carbon–hydrogen bond. The CF3 group forms alkyl interactions with Ile255 at 5.12 Å, Leu312 at 5.08 Å, and Val340 at 5.12 Å, while a pi-alkyl interaction is observed with Phe290 at 5.43 Å. The benzene ring of the ligand interacts with Val340 through pi-alkyl interactions at 5.15 and 5.40 Å and with Ala213 at 4.38 Å. Additionally, Glu342 establishes a pi–anion interaction with the benzene ring at 3.70 Å and a carbon–hydrogen bond with a carbon atom of the ligand at 3.69 Å (Table 3, Figure 13).

**Figure 13:**
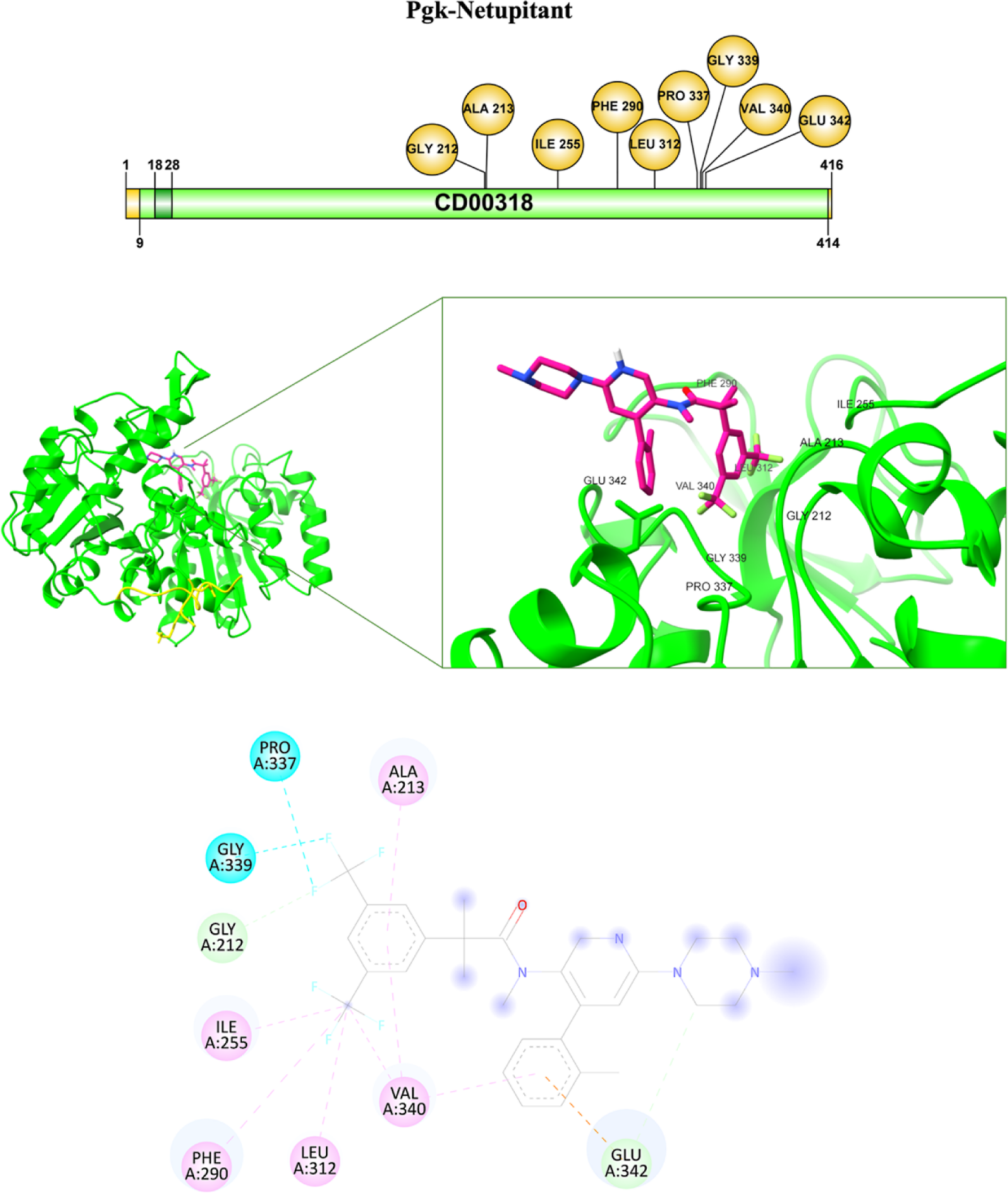
Pgk–Netupitant interaction: The binding of the Pgk-ligand complex is shown with the interaction on the specific amino acids.

Pgk belongs to a superfamily of enzymes that catalyze the transfer of the γ-phosphate group of ATP to the serine, threonine, tyrosine, or histidine amino acid residues of the target protein. These proteins have three main domains, called SH1, SH2, and SH3. Their catalytic site is located in the SH1 domain, this domain has a consensus sequence seven residues upstream of the first Gly in the consensus Gly-xxx-Gly-xxx-xxx-xxx-Gly.^69^ The SH1 domain also contains the region where the nucleotide and substrate protein bind, as well as the transfer of the phosphate group.^70^ The SH2 domain is where the recognition of the consensus sequence and the interaction with the substrate protein takes place. The SH3 domain is responsible for protein localization in the cell.^71^ The difference in the binding of the five antifungals to Pgk of *C. glabrata* is possibly because this protein, like other orthologous proteins in other organisms, has several ligand and cofactor binding sites. These analyses allow us to elucidate that, in the case of fluconazole, even though it has a greater number of interactions with Pgk, these interactions do not favor the interaction between the enzyme and this antifungal agent, since eight of the interactions are outside the enzyme’s catalytic site. Thus, only one of the interactions between fluconazole and Pgk (Ala213 residue) is found in the SH1 domain. Although this interaction is found in the SH1 domain (the domain where the catalytic site is located), it is insufficient to inhibit the enzyme (Table 3, Figure 11). It has been reported in Pgk from *Thermotoga maritima* that the catalytic site residues are conserved, but Lys-197, which is located at the C-terminus, closes this domain to the N-terminus. Also Lys-197 allows the transfer of the phosphate group from the donor to the ADP acceptor.^29^ It can be seen that residues, such as Ala213, Val340, and Asp373 (Table 3, Figure 11), are common in the interactions, which follows that these amino acids are more involved or critical in the binding of Pgk to ligands, which is interesting due to the differences in their chemical nature; Ala213 and Val340 are nonpolar residues that are involved in hydrophobic interactions, forming a stable environment inside the protein contributing to an environment that attracts and stabilizes nonpolar ligands as are all the molecules evaluated. Asp373, being polar and negatively charged, can form hydrogen bonds that strengthen the interaction. A study of pig muscle Pgk identified 3 arginine residues (at positions 65, 122, and 170) that allow interactions with the oxygen atoms of the substrate through hydrogen bridges.^72^ Interestingly, the participation of Arg122 with an oxygen atom of amphotericin B (Table 3, Figure 9) through a hydrogen bridge is observed, which corroborates the importance of this residue in establishing a stable complex. Furthermore, it has been reported in studies with Pgk from *Bacillus stearothermophilus* that Mg^2+^ allows the stabilization of the transition state of the phosphoryl group. Except residue Asp352, most of the residues are at a distance between 2.27 and 2.29 Å from Mg^2+^. It has been suggested that this ion is key for the catalytic activity of Pgk;^73^ so that, according to the in silico analysis performed, an interaction between amphotericin B and Mg^2+^can be observed with a distance of 2.58 Å, which, added to the interaction with the Arg122 residue, would suggest a better interaction which, in turn, is reflected in the better affinity energy reported (Table 2, 3, Figure 9) since this antifungal is also the only molecule without hydrophobic alkyl or pi-alkyl interactions. In the case of nystatin, the enzyme binding data showed that there is a shorter distance between Pgk and this antifungal agent (Table 3, Figure 10). This favors interaction and affinity, allowing for a greater inhibition of the enzyme with this drug. This enzyme has already been proposed as a therapeutic target against *T. brucei*.^34,35^ Pgk has also been identified on the cell surface of bacteria of the genus *Streptococcus*, where it participates in the binding of the pathogen to host proteins.^38^ For nilotinib and netupitant, several interactions occur with residues located in or near the SH1 domain, including Ala213 and Val340, which have been identified as key for ligand binding. Nilotinib forms hydrogen and pi-alkyl bonds with residues that may contribute to complex stability (Table 3, Figure 12), while netupitant shows halogen and pi–anion interactions involving Glu342 and other nonpolar residues (Table 3, Figure 13). These interactions, both in the number and in strategic localization, suggest a higher theoretical potential to interfere with Pgk activity compared to the standard antifungals. Thus, our results allow us to propose Pgk from *C. glabrata* as a therapeutic potential target against candidiasis.

### 3.7 Enzymatic Activity of Pgk with Amphotericin B, Nystatin, Fluconazole, Nilotinib, and Netupitant

In order to experimentally verify whether these compounds interact with the enzyme, we carried out the enzymatic activity assays of Pgk in the presence of each of the three selected antifungals (amphotericin B, nystatin, and fluconazole) as well as the new compounds nilotinib and netupitant. Thus, enzyme kinetic experiments were performed with pure Pgk to determine the canonical kinetics of the enzyme and whether there is any effect when any antifungal is added. After selecting the kinetic parameters of Pgk from *C. glabrata*, the following *V*_max_ and *K*_m_ values were obtained: amphotericin B (0.18 and 0.45), nystatin (0.17 and 0.36), fluconazole (0.18 and 0.39), nilotinib (0.19 and 0.78), and netupitant (0.19 and 0.63), respectively (Table 4).

**Table 4:** Kinetic Parameters of *Candida glabrata* Pgk with Its Substrate and with the Addition of the Antifungals

Pgk	*V*_max_	*K*_m_
without compounds	0.19	0.36
amphotericin B	0.18	0.45
nystatin	0.17	0.36
fluconazole	0.18	0.39
nilotinib	0.19	0.78
netupitant	0.19	0.63

As shown in Table 4, in the canonical kinetics a Michaelis–Menten type was obtained (Figure 14A), which coincides with reports of this enzyme in other species, such as Pgk from *Pisum sativum* L.^74^

**Figure 14:**
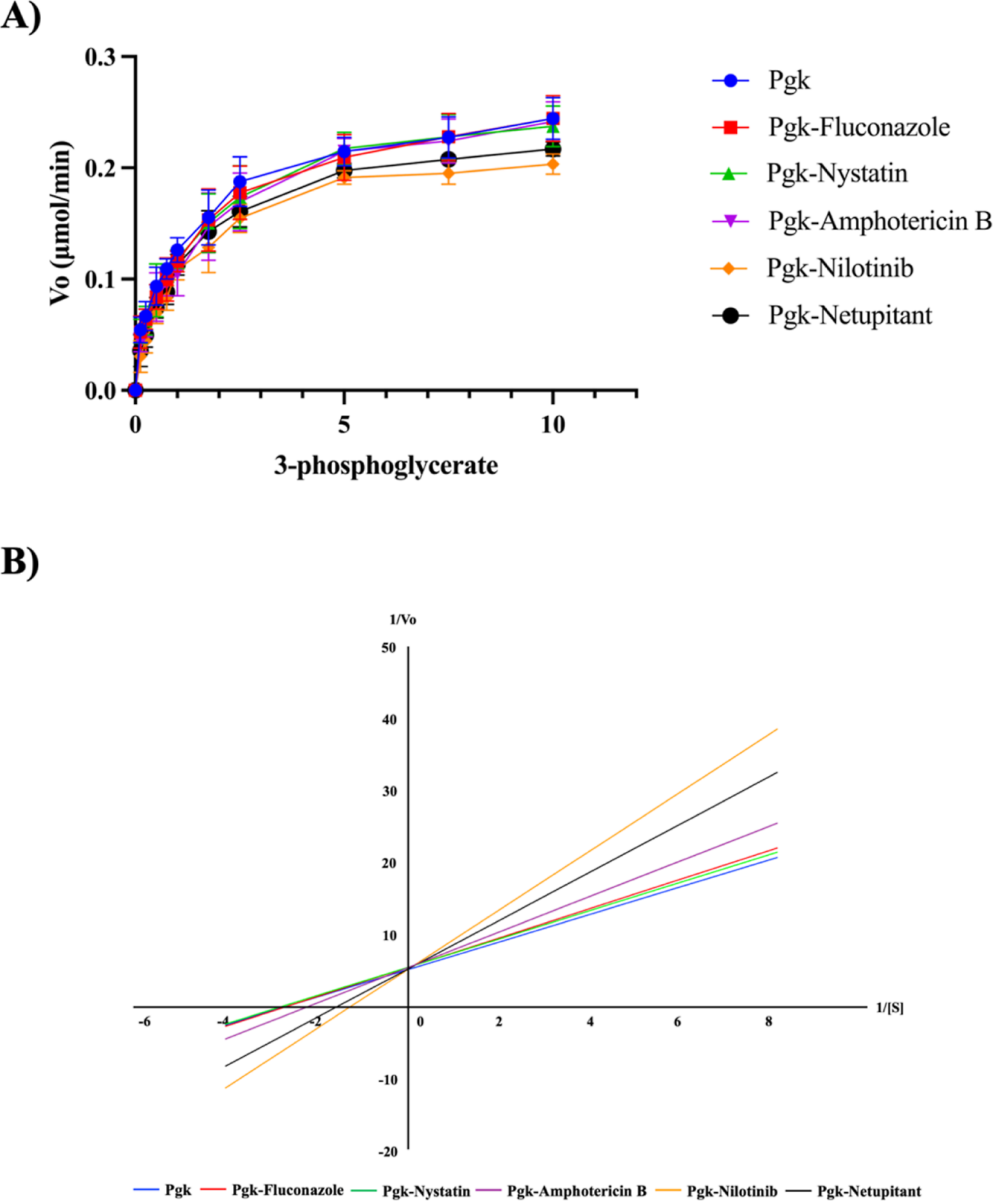
Pgk enzyme kinetics graph with its substrate and with the addition of the evaluated molecules (A). Lineweaver–Burk plot (B).

This graph shows that there are no significant differences in kinetics when the enzyme is with its substrate and the antifungals amphotericin B, nystatin, or fluconazole are added. Although the ANOVA statistical analysis (*p* value: 0.9752) and Dunnett’s test indicate that there is no significant difference, the determination of the kinetic parameters *V*_max_ and *K*_m_ was performed using the Lineweaver–Burk plot (Figure 14B) because it amplifies the differences in the reaction rates at low substrate concentrations and because this allows obtaining a more accurate estimation of the mentioned parameters from the experimental data. In addition, it facilitates the comparison between different enzymes and experimental conditions. Thus, it can be observed that although minimal there is a slight decrease in the *V*_max_ and an increase in the *K*_m_ value, which would indicate that the affinity of the enzyme for the substrate decreases discretely, which may be due to the possible interaction caused by the molecules upon coupling with Pgk. In contrast, nilotinib and netupitant showed increased *K*_m_ values (0.78 and 0.63, respectively) compared to amphotericin B, nystatin, and fluconazole. These results may indicate a differential interaction pattern compared to conventional antifungals. Previous studies have reported that nilotinib and netupitant, originally developed as anticancer drugs, exhibit measurable inhibitory effects on the growth of *C. albicans* and *C. glabrata*.^68^ This antifungal activity was consistent across concentrations, suggesting a robust biological effect.

## 4 Conclusions

IC is the leading cause of high morbidity and mortality rates in immunocompromised and hospitalized patients, with *C. glabrata* being one of the main species of this genus causing this disease. Although several antifungal drugs against IC have been available for decades, it is essential to develop new drugs due to the increasing resistance to the antifungals used against this disease. In drug design, it is essential to identify therapeutic targets in the pathogen. In the case of *C. glabrata*, our working group identified Pgk as a possible antifungal target. The first step was to elucidate its three-dimensional structure, which would allow drugs to be precisely tailored to the binding sites of Pgk with optimal binding affinity and specificity. In this work, we presented for the first time the three-dimensional structure of Pgk resolved by SAXS. In addition, in order to evaluate its potential as a therapeutic target, molecular docking studies and enzyme activity assays were performed with pure Pgk using known antifungals, such as amphotericin B, nystatin, and fluconazole and with two new plausible drugs such as nilotinib and netupitant. Our results showed some similarities and differences with orthologous Pgk proteins from other organisms, which was to be expected, as Pgk has been observed to have evolved across the kingdoms of life. Molecular docking studies showed that Pgk interacts with all the compounds tested. In the case of enzyme activity assays with amphotericin B, nystatin, fluconazole, nilotinib, and netupitant, an increased Km value was found for nilotinib and netupitant compared to those of the other antifungals tested. These results indicate a pattern of differential interactions compared to that of conventional antifungals. This antifungal activity was consistent across all concentrations, suggesting a robust biological effect. Our data reveal kinetic trends consistent with their predicted interactions in coupling simulations. These results support the potential of Pgk as a therapeutic target and provide a basis for the rational design of new molecules that could modulate its activity in future studies. In this regard, our research group is working on the design and development of new chemical molecules as potential antifungals against this protein.

## Supplementary Material



## Notes

Theoretical Ramachandran plot for the protein Pgk as well as experimental gel electrophoresis data for the recombinant protein in *E. coli* BL21 (PDF)
