# Supplementary material for: Integrative Structural Characterization of Candida glabrata Phosphoglycerate Kinase by Small-Angle X‑ray Scattering and AlphaFold: Implications for Therapeutic Targeting in Candidiasis
Source: ACS Omega. 2026 Jan 21;11(4):6628–46. doi: 10.1021/acsomega.5c11751 (PMC12878761; doi:10.1021/acsomega.5c11751)
Supplement: Supplementary file 1 [file ao5c11751_si_001.pdf]

Integrative Structural Characterization of *Candida glabrata* Phosphoglycerate Kinase by SAXS and AlphaFold: Implications for Therapeutic Targeting in Candidiasis

Mayra Cuéllar-Cruz[a*], Edson E. Maqueda-Cabrera[a], Dritan Siliqi[b], Abel Moreno[c*]

[a]*Departamento de Biología, División de Ciencias Naturales y Exactas, Campus Guanajuato, Universidad de Guanajuato, Noria Alta S/N, Col. Noria Alta, C.P. 36050, Guanajuato, Guanajuato, México.*
[b]*Istituto di Cristallografia, Consiglio Nazionale delle Ricerche, Via G. Amendola 122/O, 70126 Bari, Italy.*
[c]*Instituto de Química, Universidad Nacional Autónoma de México, Av. Universidad 3000, Ciudad Universitaria, Ciudad de México, 04510. México.*

Supporting information

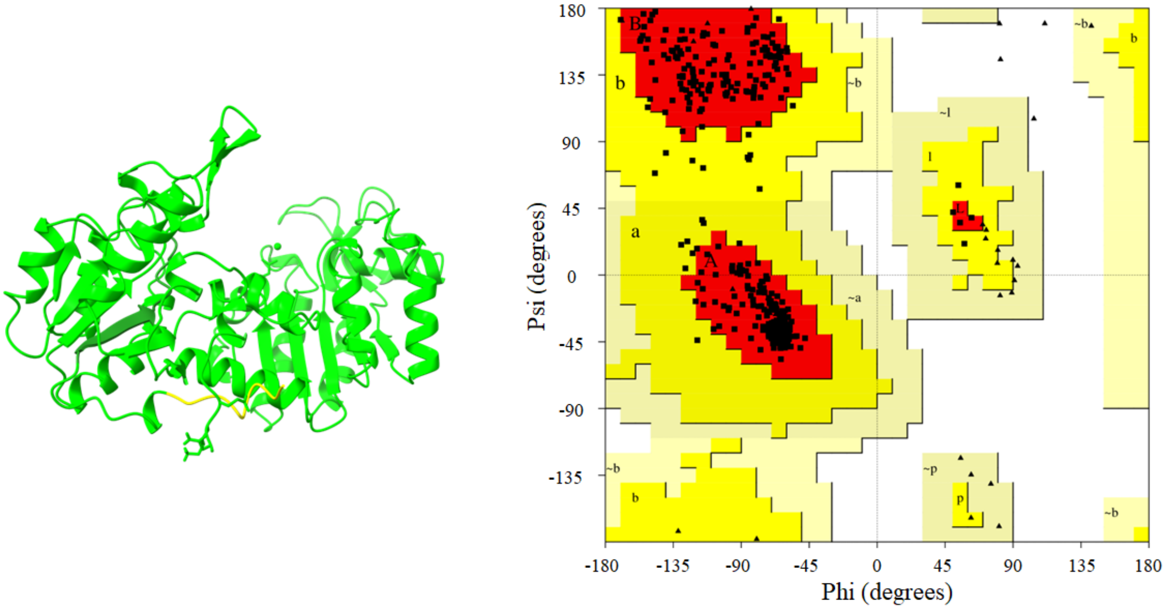

Figure S1. Ramachandran plots of Pgk, it shows the distribution of these angles in the highly stable and common FR favored regions, the less common AR allowed region, the rare but possible GR generous regions and the DR unfavorable regions.

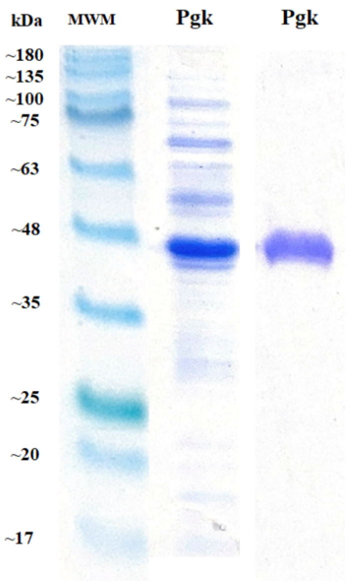

Figure S2. Expression assay of recombinant Pgk protein in *Escherichia coli* BL21 (DE3). Lane 1: Molecular weight markers. Lane 2: Cell fraction collected with IPTG. Lane 3: Pure Pgk.
